# Supplementary material for: Long-term drought and risk of infant mortality in Africa: A cross-sectional study
Source: PLoS Med. 2025 Jan 31;22(1):e1004516. doi: 10.1371/journal.pmed.1004516 (PMC11785314; doi:10.1371/journal.pmed.1004516)
Supplement: S1 Method — (DOCX) [file pmed.1004516.s002.docx]

**S1 Method** Model specification of main and secondary analysis

We used an extended Cox proportional hazards regression model with time-dependent covariates to examine the association between long-term drought and risk of infant mortality:

$$h_{i}\left( t \right)=h_{0}\left( t \right)\times\exp[\beta_{1}x_{i1}(t)+{x_{i2}^{T}\beta}_{2}+\varphi_{i}^{T}\gamma+\omega_{c(i)}]$$

where $h_{i}\left( t \right)$ is the hazard function for mother/infant pair *i* at month *t*, *h*_0_(*t*) is the unspecified baseline hazard at month *t*; *x_i_*_1_(*t*) represents a time-varying indicator for drought exposure, either a binary variable for any drought (0 for no drought and 1 for any drought) or a three-level categorical variable for mild and severe drought (0 for no drought, 1 for mild drought, and 2 for severe drought); *x_i_*_2_ is a vector of time-invariant covariates, including child’s sex, area of residence, mother’s education, and wealth quintile; *φ_i_* denotes a vector of categorical birth month and a natural cubic spline of birth year with three degrees of freedom to adjust for seasonality and long-term trend, respectively; and *ω_c(i)_* is a random intercept for a composite indicator for country and survey cluster $c(i)$.

We employed generalized linear mixed-effects logistic regression models to quantify the association between monthly drought exposure before or after birth and risk of infant mortality within each month before one year of age:

$$\ln\left( \frac{P_{i}}{1-P_{i}} \right)=\beta_{0}+\beta_{1}x_{i1}+{x_{i2}^{T}\beta}_{2}+\varphi_{i}^{T}\gamma+\omega_{c(i)}$$

where *P_i_* is the probability of infant mortality during a specific month/period for infant *i*, and the new *x_i_*_1_ represents a monthly indicator of drought exposure or the number of drought months experienced during pregnancy or infancy. The remaining terms have been previously described.
