# Supplementary material for: Long-term drought and risk of infant mortality in Africa: A cross-sectional study
Source: PLoS Med. 2025 Jan 31;22(1):e1004516. doi: 10.1371/journal.pmed.1004516 (PMC11785314; doi:10.1371/journal.pmed.1004516)
Supplement: S1 Table — (DOCX) [file pmed.1004516.s003.docx]

**S1 Table** Descriptive statistics for the number of drought months experienced by all included children by exposure window, year of birth, climate zone, and drought severity

|  | **Mean (SD)** | **Minimum** | **Median** | **Maximum** | **Interquartile range** |
| --- | --- | --- | --- | --- | --- |
| **Overall** |  |  |  |  |  |
| Any drought | 7.1 (7.3) | 0 | 5 | 21 | 0–13 |
| Mild drought | 5.1 (5.6) | 0 | 3 | 21 | 0–9 |
| Severe drought | 2.0 (4.2) | 0 | 0 | 21 | 0–1 |
| **Before birth** |  |  |  |  |  |
| Any drought | 3.3 (3.8) | 0 | 1 | 9 | 0–8 |
| Mild drought | 2.4 (3.0) | 0 | 1 | 9 | 0–5 |
| Severe drought | 0.9 (2.3) | 0 | 0 | 9 | 0–0 |
| **After birth** |  |  |  |  |  |
| Any drought | 3.8 (4.5) | 0 | 1 | 12 | 0–8 |
| Mild drought | 2.8 (3.6) | 0 | 1 | 12 | 0–5 |
| Severe drought | 1.0 (2.6) | 0 | 0 | 12 | 0–0 |
| **Born during 1992–2005** | |  |  |  |  |
| Any drought | 6.7 (7.3) | 0 | 4 | 21 | 0–12 |
| Mild drought | 4.7 (5.4) | 0 | 2 | 21 | 0–8 |
| Severe drought | 2.1 (4.2) | 0 | 0 | 21 | 0–2 |
| **Born during 2006–2019** | |  |  |  |  |
| Any drought | 7.3 (7.4) | 0 | 5 | 21 | 0–13 |
| Mild drought | 5.3 (5.6) | 0 | 4 | 21 | 0–9 |
| Severe drought | 2.0 (4.2) | 0 | 0 | 21 | 0–1 |
| **Tropical zone** |  |  |  |  |  |
| Any drought | 7.7 (7.5) | 0 | 6 | 21 | 0–14 |
| Mild drought | 5.5 (5.7) | 0 | 4 | 21 | 0–10 |
| Severe drought | 2.1 (4.3) | 0 | 0 | 21 | 0–2 |
| **Temperate zone** |  |  |  |  |  |
| Any drought | 5.6 (6.6) | 0 | 2 | 21 | 0–10 |
| Mild drought | 4.1 (5.0) | 0 | 2 | 21 | 0–7 |
| Severe drought | 1.4 (3.5) | 0 | 0 | 21 | 0–0 |
| **Dry zone** |  |  |  |  |  |
| Any drought | 6.9 (7.4) | 0 | 4 | 21 | 0–13 |
| Mild drought | 4.8 (5.4) | 0 | 3 | 21 | 0–9 |
| Severe drought | 2.1 (4.4) | 0 | 0 | 21 | 0–2 |
