# Supplementary material for: Long-term drought and risk of infant mortality in Africa: A cross-sectional study
Source: PLoS Med. 2025 Jan 31;22(1):e1004516. doi: 10.1371/journal.pmed.1004516 (PMC11785314; doi:10.1371/journal.pmed.1004516)
Supplement: S2 Table — SPEI: standardized precipitation evapotranspiration index. (DOCX) [file pmed.1004516.s004.docx]

**S2 Table** Number of infant deaths (among 850,924 children) during each month within one year of age and number of months for SPEI-24 any drought experienced before and after birth. SPEI: standardized precipitation evapotranspiration index.

| Month | Missing/ excluded (%) | Infant deaths | | | | | |
| --- | --- | --- | --- | --- | --- | --- | --- |
|  |  | Yes | | | No | | |
|  |  | *N* (%) | Average months before birth (SD) | Average months after birth (SD) | *N* (%) | Average months before birth (SD) | Average months after birth (SD) |
| 27 days | 0^†^ | 26,704  (3.1) | 3.3  (3.8) | 0.4  (0.5) | 824,220  (96.9) | 3.3  (3.8) | 3.9  (4.6) |
| 2 months | 43,005  (5.1) | 3179  (0.4) | 3.2  (3.7) | 0.7  (0.9) | 804,740  (94.6) | 3.3  (3.8) | 4.0  (4.6) |
| 3 months | 62,441  (7.3) | 2786  (0.3) | 3.4  (3.8) | 1.1  (1.4) | 785,697  (92.3) | 3.3  (3.8) | 4.1  (4.6) |
| 4 months | 81,026  (9.5) | 2744  (0.3) | 3.2  (3.7) | 1.4  (1.8) | 767,154  (90.2) | 3.3  (3.8) | 4.1  (4.6) |
| 5 months | 99,788  (11.7) | 2007  (0.2) | 3.4  (3.8) | 1.8  (2.2) | 749,129  (88.0) | 3.3  (3.8) | 4.2  (4.7) |
| 6 months | 117,385  (13.8) | 1755  (0.2) | 3.3  (3.8) | 2.2  (2.6) | 731,784  (86.0) | 3.3  (3.8) | 4.2  (4.7) |
| 7 months | 134,988  (15.9) | 2392  (0.3) | 3.5  (3.8) | 2.7  (3.0) | 713,544  (83.9) | 3.4  (3.8) | 4.3  (4.7) |
| 8 months | 152,750  (18.0) | 1976  (0.2) | 3.6  (3.8) | 3.2  (3.4) | 696,198  (81.8) | 3.4  (3.8) | 4.3  (4.8) |
| 9 months | 169,653  (19.9) | 1920  (0.2) | 3.3  (3.8) | 3.4  (3.7) | 679,351  (79.8) | 3.4  (3.8) | 4.3  (4.8) |
| 10 months | 185,829  (21.8) | 2109  (0.2) | 3.4  (3.8) | 3.8  (4.1) | 662,986  (77.9) | 3.4  (3.8) | 4.4  (4.8) |
| 11 months | 201,773  (23.7) | 1175  (0.1) | 3.6  (3.8) | 4.2  (4.5) | 647,976  (76.1) | 3.4  (3.8) | 4.4  (4.8) |
| 12 months | 216,668  (25.5) | 1210  (0.1) | 3.4  (3.8) | 4.4  (4.9) | 633,046  (74.4) | 3.4  (3.8) | 4.4  (4.8) |

^†^ A total of 9952 infants were excluded from the original dataset because they were alive and less than 27 days old at the time of interview (i.e., the original total number of children was 860,876)
