# Supplementary material for: Long-term drought and risk of infant mortality in Africa: A cross-sectional study
Source: PLoS Med. 2025 Jan 31;22(1):e1004516. doi: 10.1371/journal.pmed.1004516 (PMC11785314; doi:10.1371/journal.pmed.1004516)
Supplement: S3 Table — (DOCX) [file pmed.1004516.s005.docx]

**S3 Table** Association between long-term drought and risk of infant mortality in various sensitivity analyses

|  | **Estimate** | **Standard error** | **Lower bound** | **Upper bound** |
| --- | --- | --- | --- | --- |
| **Main analysis** |  |  |  |  |
| Any drought | 1.019 | 0.010 | 0.998 | 1.040 |
| Mild drought | 1.011 | 0.011 | 0.988 | 1.034 |
| Severe drought | 1.040 | 0.016 | 1.008 | 1.074 |
| **Rainfall adjusted** |  |  |  |  |
| Any drought | 1.019 | 0.010 | 0.998 | 1.040 |
| Mild drought | 1.011 | 0.011 | 0.988 | 1.034 |
| Severe drought | 1.040 | 0.016 | 1.008 | 1.074 |
| **Birth month removed** |  |  |  |  |
| Any drought | 1.019 | 0.010 | 0.999 | 1.040 |
| Mild drought | 1.011 | 0.011 | 0.988 | 1.034 |
| Severe drought | 1.041 | 0.016 | 1.009 | 1.075 |
| **Complementary log-log regression** |  |  |  |  |
| Any drought | 1.013 | 0.007 | 0.998 | 1.027 |
| Mild drought | 1.003 | 0.008 | 0.988 | 1.019 |
| Severe drought | 1.038 | 0.011 | 1.015 | 1.061 |
| **Postnatal period only** |  |  |  |  |
| Any drought | 1.029 | 0.015 | 0.999 | 1.060 |
| Mild drought | 1.024 | 0.017 | 0.991 | 1.058 |
| Severe drought | 1.041 | 0.024 | 0.994 | 1.091 |
