# Supplementary material for: Long-term drought and risk of infant mortality in Africa: A cross-sectional study
Source: PLoS Med. 2025 Jan 31;22(1):e1004516. doi: 10.1371/journal.pmed.1004516 (PMC11785314; doi:10.1371/journal.pmed.1004516)
Supplement: S4 Table — (DOCX) [file pmed.1004516.s006.docx]

**S4 Table** Estimates and uncertainties of drought exposure before and after birth when including gestational drought exposure in the sensitivity analysis of postnatal period only

|  | **Estimate** | **Standard error** | **Lower bound** | **Upper bound** |  |  |  |  |
| --- | --- | --- | --- | --- | --- | --- | --- | --- |
| Number of drought months before birth |  |  |  |  |  |  |  |  |
| Any drought model | 1.0063 | 0.0023 | 1.0018 | 1.0108 |  |  |  |  |
| Mild & severe drought model^†^ | 1.0062 | 0.0023 | 1.0017 | 1.0107 |  |  |  |  |
| Drought exposure after birth |  |  |  |  |  |  |  |  |
| Any drought | 1.0054 | 0.0173 | 0.9719 | 1.0400 |  |  |  |  |
| Mild drought | 1.0035 | 0.0183 | 0.9682 | 1.0402 |  |  |  |  |
| Severe drought | 1.0114 | 0.0141 | 0.9610 | 1.0644 |  |  |  |  |
| Any drought month before birth | | | | |  |  |  |  |
| Any drought model | 1.0402 | 0.0166 | 1.0068 | 1.0747 |  |  |  |  |
| Mild & severe drought model | 1.0396 | 0.0167 | 1.0062 | 1.0742 |  |  |  |  |
| Drought exposure after birth |  |  |  |  |  |  |  |  |
| Any drought | 1.0122 | 0.0166 | 0.9798 | 1.0457 |  |  |  |  |
| Mild drought | 1.0088 | 0.0179 | 0.9741 | 1.0448 |  |  |  |  |
| Severe drought | 1.0219 | 0.0250 | 0.9729 | 1.0733 |  |  |  |  |

^†^ In this model, mild and severe drought conditions were represented by a three-level categorical indicator (no drought, mild drought, and severe drought).
