# Supplementary material for: Long-term drought and risk of infant mortality in Africa: A cross-sectional study
Source: PLoS Med. 2025 Jan 31;22(1):e1004516. doi: 10.1371/journal.pmed.1004516 (PMC11785314; doi:10.1371/journal.pmed.1004516)
Supplement: S1 Fig — Climate variables include maximum and minimum temperatures, dew point temperature, wind speed, solar radiation, and air pressure. SPEI: standardized precipitation evapotranspiration index. (DOCX) [file pmed.1004516.s007.docx]

**S1 Figure** Illustration of calculation of SPEI at a timescale of 24 months for a given month from the start of pregnancy through death or the 12th month after birth. Climate variables include maximum and minimum temperatures, dewpoint temperature, wind speed, solar radiation, and air pressure. SPEI: standardized precipitation evapotranspiration index.

**
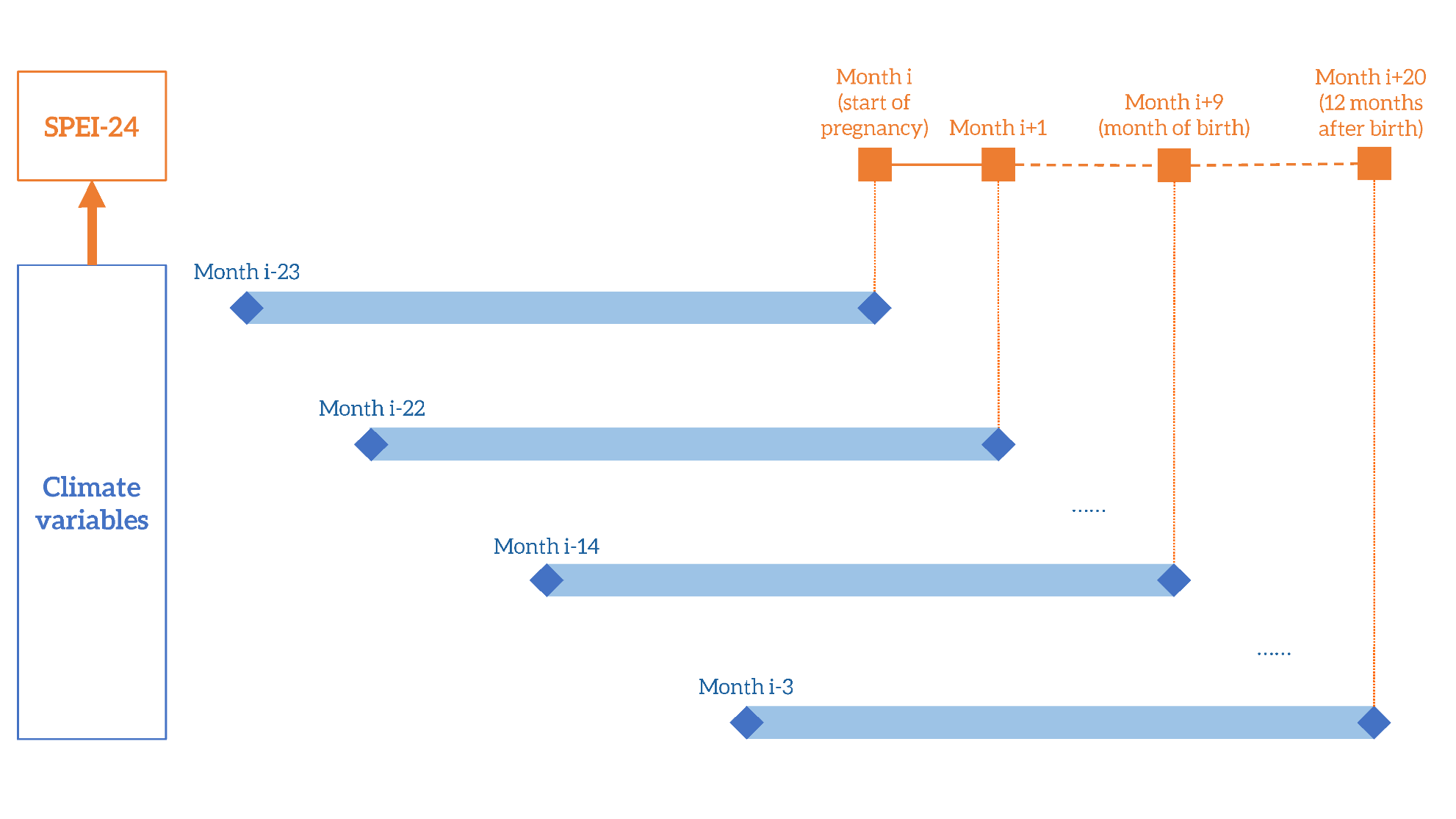
**
