# Supplementary material for: Long-term drought and risk of infant mortality in Africa: A cross-sectional study
Source: PLoS Med. 2025 Jan 31;22(1):e1004516. doi: 10.1371/journal.pmed.1004516 (PMC11785314; doi:10.1371/journal.pmed.1004516)
Supplement: S2 Fig — (DOCX) [file pmed.1004516.s008.docx]

**S2 Figure** Associations between neonatal and post-neonatal mortality and drought exposure by month of pregnancy

**
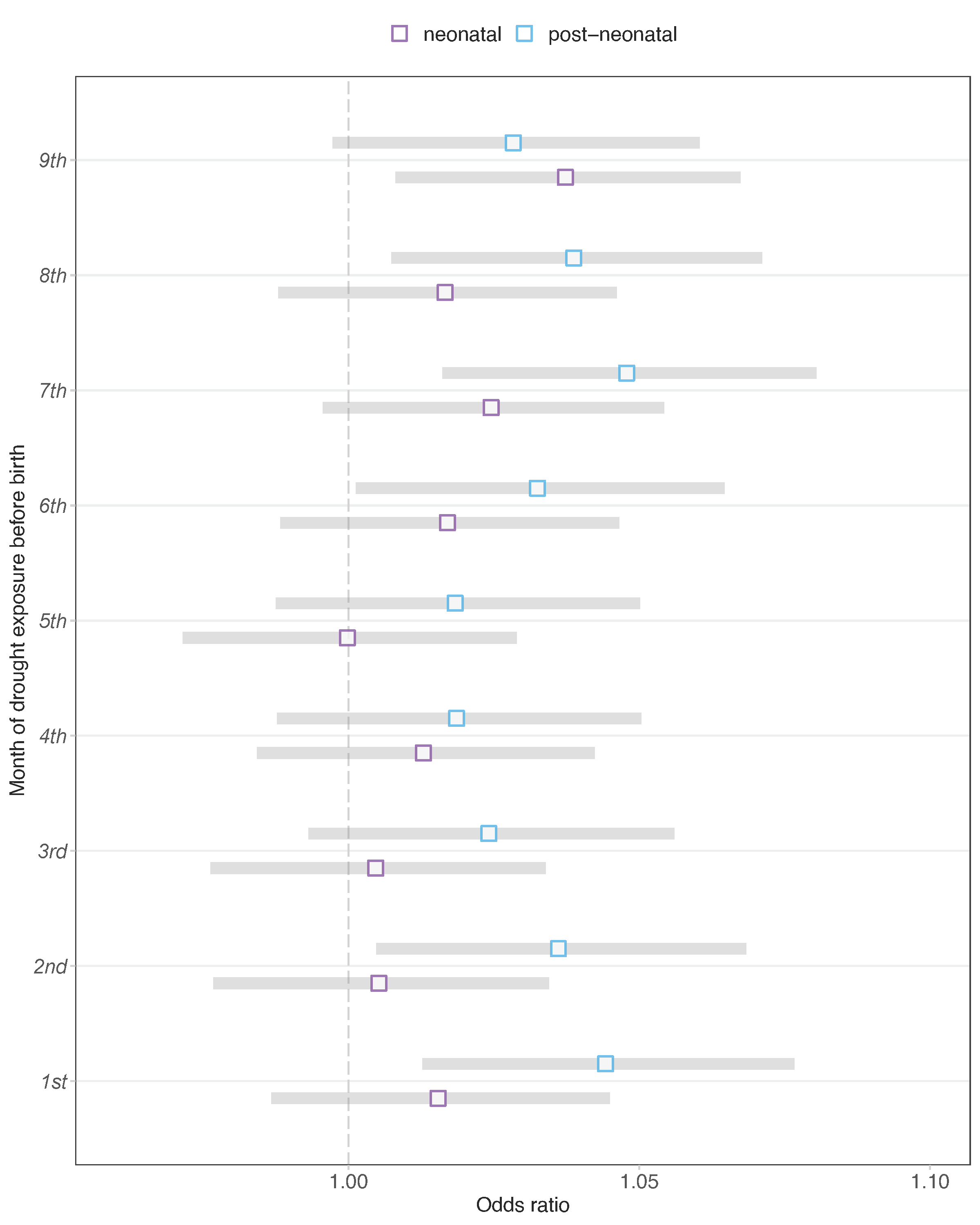
**
