# Supplementary material for: Long-term drought and risk of infant mortality in Africa: A cross-sectional study
Source: PLoS Med. 2025 Jan 31;22(1):e1004516. doi: 10.1371/journal.pmed.1004516 (PMC11785314; doi:10.1371/journal.pmed.1004516)
Supplement: Attachment — Submitted filename: wang.pdf [file pmed.1004516.s012.pdf]

This paper examines the association between drought and infant mortality in Africa.

Background

I was asked to look at this submission to arbitrate on a difference of opinion between the statistical reviewer and the authors. I have been provided with copies of the submissions, the reviewer comments, and author rebuttals.

I have not attempted to re–review the current submission since I was not asked to and, in any event, at this late stage it would be inappropriate. I have, however, read the current submission since my primary goal when performing a statistical review is to ascertain whether the science and the statistics match.

What is the issue?

The scientific question

Just so we all agree here we can do no better than to quote from page 7

> To fill these research gaps, by using data from 34 African countries and developing a complex drought indicator, we aimed to 1) examine the association between drought and risk of infant mortality; 2) investigate whether the association varies between neonatal and postneonatal deaths; and 3) explore the potential delayed effect of drought exposure on infant mortality.

Infant mortality is implicitly defined as from birth to 364 days which for some purposes is split into neonatal (up to 27 days of life) and later (28 to 364 days) (page 9).

The statistical issue

There is no dispute that the Cox model is appropriate and also the logistic model as outlined in S1 Method. The issue turns on when time is taken to start. The authors start time at conception. Since that is unobservable they impute it as occurring nine months before birth. Obviously this rests on assumptions but you have to make some assumptions in life to get anywhere and this imputation has not been disputed. The statistical reviewer by contrast takes the view that since the outcome is infant mortality the time should start at birth since it is only then that the child is at risk of

infant mortality. My initial response would have been that I agreed with the reviewer as I cannot see why one would want to include time when the participants were not at risk of the event in a time to event model. The authors have responded that

> We apologize again for making contradictory statements in our response letters. However, we argue that maternal exposure to drought during pregnancy is potentially associated with infant mortality through the mother, similar to other environmental exposures as stated before. In this sense, it is crucial for us to include the pregnancy period in our study. This is the primary and foremost reason why we used the start of pregnancy as the beginning of our analysis. Excluding the prenatal period would significantly jeopardize the quality of our study and inevitably raise questions about missing this critical exposure window. Furthermore, as we mentioned in our previous response letter, we would like to keep drought from the start of pregnancy through infant deaths as our exposure also because the exploration of the potential delayed effect of drought exposure during pregnancy is one of the highlights of our study.

I am not an expert in human development but it seem obvious that adverse circumstances during pregnancy would affect the mother–to–be's chances of producing a thriving infant. Those circumstances seem to me to be an exposure, in the terminology of the equation in S1 Method they would be in the x_{i2}. As I read the authors' rebuttal quoted above it seems to me that although they agree that it is an exposure they seem unwilling that it should be treated as such. As it stands I do not see how the goal of assessing its effect is being met by the authors' analysis.

The sensitivity analyses

The authors have fitted the model as suggested by the reviewer as one of their sensitivity analyses and report it on page 12.

> In addition, when excluding drought exposure during pregnancy, the association with severe drought was found to be non-significant (Figure S3).

This is insufficient. Just showing a graph without any numerical values does not allow us to make a proper comparison between the models. Looking at figure S3 it seems (using a ruler) that the confidence intervals are somewhat wider. My intuition here would be that excluding irrelevant time periods

might have given more precise estimates, not less. The reviewer had already suggested this.

The conclusions

In the abstract we read

> Compared to children who did not experience drought, any drought exposure was associated with an increased risk of infant mortality (hazard ratio [HR]: 1.02, 95% confidence interval [CI] [1.00, 1.04], p=0.072).

Given the findings of the two models this seems to me to be far too strong. The authors are adopting the standard null hypothesis significance test (NHST) model and state on page 10 that their critical α is 0.05. Given that the statement in the abstract should read "Compared to children who did not experience drought, we were unable to find evidence that any drought exposure was associated with an increased risk of infant mortality (hazard ratio [HR]: 1.02, 95% confidence interval [CI] [1.00, 1.04], p=0.072)." In fact given the narrowness of the confidence intervals one could stretch a point and state there is evidence of no effect. That would also make clear that both the authors' preferred analysis and that of the statistical reviewer concur in the scientific finding. The discordance of the finding about severe drought should remain. Some corresponding changes would be needed elsewhere. In particular the title 'The drier, the deadlier' is not what was found by the authors.

Recommendations

- Provide full details of the sensitivity models (ideally all of them not just the one using time since birth).
- Change the abstract as I have suggested so as to comply with NHST.
- Since the two models agree on all the substantive issue except severe drought there is no pressing need to swap the roles of primary analysis and sensitivity analysis.

Obviously the fact that the authors did not find any evidence of effect has no relevance on the decision to publish or not.

Potential conflicts of interest (CoI)

For the record I do not know the identity of the statistical reviewer which avoids any possible CoI there. I can see no CoIs with the authors either.

In the interests of full disclosure I should perhaps state that I have collaborated in studies in sub–Saharan Africa including one on maternal mental health in the perinatal period. I cannot imagine any CoIs with my current task arising as a result.

Michael Dewey
