# Supplementary material for: Long-term drought and risk of infant mortality in Africa: A cross-sectional study
Source: PLoS Med. 2025 Jan 31;22(1):e1004516. doi: 10.1371/journal.pmed.1004516 (PMC11785314; doi:10.1371/journal.pmed.1004516)
Supplement: Attachment — Submitted filename: wangrereview.pdf [file pmed.1004516.s014.pdf]

This paper examines the association between drought and infant mortality in Africa. I will not repeat here the background to my previous review.

Conclusions in the abstract

The authors state

> However, when excluding drought exposure during pregnancy, the association with severe drought was found to be non-significant.

and

> Exposure to long-term severe drought was associated with increased infant mortality risk in Africa.

These contradict one another. The authors have fitted two different models, their preferred one and the one suggested by the statistical reviewer. Each gives three views of the data: overall, severe and not severe. Only one of the six showed a difference. The conclusion is still too strong.

Comparing the models

The authors state in their rebuttal.

> This further demonstrates the importance of the inclusion of the pregnancy period in the analysis, which gave more precise estimates, not less.

Exactly. That is why in my review I stated *Those circumstances seem to me to be an exposure, in the terminology of the equation in S1 Method they would be in the* x_{i2}.

The authors have not, as far as I can see, included this. If they truly believe exposure in pregnancy is important they should have included it in the sensitivity analysis as I suggested, as an exposure, an x_{i2} and then reported the coefficient.

Summary

The authors seem very reluctant to change anything in the light of the very real concerns that I and the other statistician raised.

Michael Dewey
