# Supplementary material for: Long-term drought and risk of infant mortality in Africa: A cross-sectional study
Source: PLoS Med. 2025 Jan 31;22(1):e1004516. doi: 10.1371/journal.pmed.1004516 (PMC11785314; doi:10.1371/journal.pmed.1004516)
Supplement: Attachment — Submitted filename: Response to reviewers_revised.docx [file pmed.1004516.s009.docx]

**Response to reviewers**

We sincerely thank all reviewers for the comments and suggestions that significantly improve this manuscript. We made major revisions to the previous draft, particularly for the Results and Discussion, to address the comments from the reviewers (blue colored) and marked down the corresponding location in the text for each response below.

**Reviewer #1**

*The authors examine the link between drought and infant mortality in Africa. Africa in general is an understudied region, particularly for infant health impacts, and there is little research globally on the links between drought and infant mortality.*

*The authors have used the SPEI to measure drought, which is a widely used index for drought, and has been linked to agricultural and other impacts, more so than SPI or precipitation-only drought indices. This therefore is an appropriate way to measure drought in this study. The authors have also considered a number of different variables which would impact on infant mortality, such as wealth, rural/urban, education, climate zone etc. The authors have also pointed out that the link between drought and infant mortality is likely indirect, and due to drought impacts on water quality, illnesses, and food supply.*

*Perhaps because the paper has already been through one round of revisions, I don't have much feedback to give, and my feedback is mostly minor editing.*

***Q1.*** *Introduction - line 103, 'studied' not 'studies'.*

**A1.** Thank you for noticing this typo. We have revised this accordingly in the manuscript (line 123 in the tracked version, line 119 in the clean version):

*“This region of the world deserves careful scrutiny, not only because the human health effects of climate change are infrequently studied among African populations, but also because it is one of the fastest growing populations, expected to be home to a quarter of the world’s population by 2050.”*

***Q2.*** *Results - paragraph 227 - 232 - the first and second sentence in this paragraph are very similar. Is this intentional?*

**A2.** Now we re-performed the subgroup analysis based on the comments from another reviewer, and we revised this paragraph as below (lines 257-260 in the tracked version, lines 243-245 in the clean version):

*“We found that the association was significantly stronger among children living in rural areas than those living in urban areas and among children living in tropical climate zones than those living in dry zones (Figure 3). We did not observe significant effect modification by other factors.”*

**Reviewer #2**

*This study investigates the impact of drought on infant mortality in 34 African countries using data from 1992 to 2019. Aiming to address the gap in understanding how environmental factors like drought contribute to infant mortality rates, which remain high despite a global decline, the study employs the standardized precipitation evapotranspiration index to measure drought intensity. The analysis uses time-varying Cox proportional hazard regression models to estimate the association between long-term drought and infant mortality during the neonatal and postnatal stages, considering the potential delayed effects of drought exposure. Results indicate that while exposure to any drought shows a non-significant increase in risk, severe drought is significantly associated with higher infant mortality, especially in the neonatal period. While the study utilizes high-quality data and a solid method for measuring drought, concerns arise regarding the statistical methods employed, particularly the calculation of the confidence intervals (CI) for the difference between subgroup effects. Additionally, considering the numerous mediated pathways between drought and infant mortality as the authors also acknowledge, it would be beneficial to investigate these pathways in detail rather than focusing solely on the distal effects of drought. This approach could potentially enhance the study's impact on policy formulation. Detailed comments are provided below.*

***Q1****. Line 71-107, Introduction: While the introduction mentions the global decline in infant mortality rates and the Sustainable Development Goals, it could benefit from a more in-depth discussion of the specific challenges and trends in infant mortality in Africa. This could include factors like healthcare access, socioeconomic conditions, and regional differences within Africa. This broader context would help readers understand the unique challenges the continent faces in reducing infant mortality.*

**A1.** Thank you for your suggestions. We added some descriptions on these associated factors to show the challenges Africa is still facing (lines 117-121 in the tracked version, lines 113-117 in the clean version):

*“In Africa, infant mortality is associated not only with various demographic and socioeconomic characteristics but also with local cultural settings and societal structural frameworks, such as differences in healthcare facilities provision between urban and rural areas, domestic healthcare expenditure, and foreign resources flowing into health,*^1,2^ *which remain challenges the continent still faces.”*

***Q2****. Line 71-107, Introduction: While the study's aim is to investigate the association between drought and infant mortality, the introduction could further discuss the potential policy and practical implications of the findings. For instance, how might the results inform public health interventions, climate adaptation strategies, or resource allocation in affected regions?*

**A2.** Thank you for your comments. These topics are not directly related to the background of our study. However, we believe that these are important implications from this study. Therefore, we added some descriptions at the end of the manuscript (lines 359-364 in the tracked version, lines 343-348 in the clean version):

*“Comprehensive engagement, planning, and preparedness should be advanced among pregnant women and children that are disproportionately affected by drought, particularly in rural and tropical settings. Understanding the association between drought and infant mortality can guide a multi-faceted approach in Africa involving early warning systems, neonatal and pediatric care infrastructure planning, community education, food security program development, and water and sanitation interventions.”*

***Q3.*** *Line 140-141, Method: "..then a normalization procedure was applied to this series to eliminate the effect of both the local season and climate patterns." It would be beneficial to briefly describe the normalization procedure in the manuscript for the reader's comprehension. Detailing this method is crucial as it underpins the study's analytical approach.*

**A3.** We have modified this statement as below (lines 159-164 in the tracked version, lines 155-160 in the clean version):

*“In order to compute the SPEI at a timescale of 24 months (referred to as SPEI-24 hereinafter) for a given month during the study period, we first generated a time series by summing the water balance over the preceding 23 months up to the current month; then we transformed this series to a normal distribution with a mean of zero and standard deviation of one to eliminate the effect of both the local season and climate patterns.”*

***Q4.*** *Line 152, Method: Is "long-term drought" the 24-month SPEI mentioned above. I think it would be better to be specific here.*

**A4.** We revised it accordingly (lines 174-176 in the tracked version, lines 170-171 in the clean version):

*“We used an extended Cox regression model with time-dependent covariates to examine the association between drought at a timescale of 24 months and risk of infant mortality.”*

***Q5.*** *Line 151-154, Method: The manuscript refers to the use of a 'proportional hazards regression model' while also allowing for 'nonproportional hazards' over time. To prevent confusion, it is imperative that the authors provide a clear explanation of the model's application, particularly how it accommodates nonproportional hazards within its framework.*

A5. We apologize for the misleading description. The time-varying Cox models have two applications. First, it can accommodate time-dependent covariates, such as the investigation of the effect of changing smoking status on cancer risk during the following-up period. In our case, monthly drought indicator was the time-dependent covariate. Second, it can also accommodate time-varying coefficients that violate the assumption of proportional hazard for the original Cox models. For example, a covariate is measured at baseline but its effect on the outcome is not constant over the follow-up time. Only the first situation applied to our study. So we modified our description of this model as (lines 176-179 in the tracked version, lines 171-175 in the clean version):

*“Previously applied in other infant death studies, this extended model allows for time-varying covariance caused by the change of a given covariate over time during the follow-up period, and estimates the effect of a time-varying variable on the survival probability at a specific time contingent upon the values of that variable at the same time.”*

In addition, we replaced all “Cox proportional hazards regression model” with “Cox regression model” throughout the manuscript to avoid confusion (lines 61, 174, and 218 in the tracked version, lines 58, 170, and 208 in the clean version).

***Q6.*** *Line 155-157, Method: "Moreover, this time dependent analysis can help prevent the immortal time bias, which is characterized by the misalignment between the start of pregnancy (time zero) and exposure." The authors suggest that the time-dependent analysis is used to prevent immortal time bias, which arises from the misalignment of pregnancy start time and exposure period. However, if gestational age is uniformly assumed to be 9 months due to data limitations, it remains unclear how the method corrects for the potential misalignment and consequent bias. Clarification on the approach used to address this specific type of bias would be beneficial for the reader's understanding.*
**A6.** Thank you so much for pointing this out. Initially our analysis was stratified as pre-birth and after-birth analyses, so we thought there would be potential immortal time bias in the after-birth analysis because the exposure started at birth. But we have dropped that analysis. There should be no such bias in the current analysis because our start of “follow-up” and start of exposure are both the start of pregnancy (time zero). Although each fetus had to survive through the entire pregnancy to be eligible to be included in the study sample, we have already acknowledged this limitation in the manuscript. In this revised manuscript, we have deleted the statement on the immortal time bias.

***Q7.*** *Line 166-170, Method: Could the subgroup analyses be conducted by incorporating the relevant variables and their interaction terms with the exposure variable (SPEI) into the model? I think it is easier to conduct the subgroup analyses this way.*

A7. Thank you for your suggestion. Based on your next comment, we now use interaction term between the binary drought indicator and each variable to test their potential effect modification. The results are now shown in the revised Figure 3. The difference between the estimates for temperate and dry zones is no longer statistically significant.

***Q8.*** *Line 170-173, Method: Also, the current method for calculating the confidence interval (CI), or more precisely the standard error (SE) of the difference, appears incorrect. The formula for the variance of the difference between two dependent random variables (A and B) should account for their covariance, as Var(A−B)=Var(A)+Var(B)−2Cov(A,B), not simply Var(A)+Var(B) which assumes independence. Given that variables A (e.g., effects of SPEI on mortality among males) and B (e.g., effects of SPEI on mortality among females) are likely not independent, the calculation of the CI might be flawed.*

A8. Thank you for your comments. We agree that the two subgroups from the same large sample might not be independent, which could bias our estimate for the variance. Now we use the P values for the interaction term between the binary drought indicator and each potential effect modifier to indicate the significance of the effect modification. We revised the manuscript accordingly (lines 190-199 in the tracked version, lines 183-189 in the clean version):

*“We then stratified the main analysis by several baseline characteristics by incorporating an interaction term between the binary indicator for any drought and each of the following factor: child’s sex (male and female), area of residence (urban and rural), mother’s education (no education and any education), household’s wealth status (lower as the 1st–2nd quintiles and higher as the 3rd–5th quintiles), year period of birth (1992–2005 and 2006–2019) and climate zone (tropical, temperate, and dry). The statistical significance of the difference between subgroups was indicated by the p-value of the interaction term.”*

**Reviewer #3**

*Thank you for the possibility to review this article. "The drier, the deadlier: The association between long-term drought and risk of infant mortality in Africa" is a research article that aims to investigate the association between drought and risk of infant mortality in Africa using DHS data for infant mortality and drought by the standardized precipitation evapotranspiration index. This is a well-executed and well-written article that will be an important contribution to the literature. First some more overarching questions and then a couple of minor comments.*

***Q1.*** *Why were no authors from Africa in the team? It would be good to have colleagues from the setting where the study is situated. Might be difficult at this stage; but wonder about the rationale behind this decision and a suggestion for future studies within this setting.*

**A1.** Thank you for your concern. You are correct that the perspectives from authors from Africa matter in terms of the implication and communication of our scientific findings. We identified a collaborator at Yale who is originally from Ghana (Ernest O. Asare) and two other collaborators in Ghana and Côte d'Ivoire (Robert A. Akum and N'datchoh E. Toure) that are among the countries in our analysis. We sent them the manuscript and they made helpful edits and insightful and valuable suggestions for the improvement of the manuscript.

***Q2.*** *Why did you decide to focus on just infant mortality and neonatal mortality. What about the under-five mortality? Did you study this as well? This data is also available in DHS.*

**A2.** We are aware of the availability of under-five mortality information in DHS datasets. As you indicated below, a previous study in Ethiopia (the only one we can find in terms of the quantitative association between drought and under-five child mortality) found no significant impact of drought on under-five mortality. Furthermore, the purpose of this study was to evaluate the association between drought exposure before and after birth and infant deaths, which would be more biologically linked to drought exposure during pregnancy than child mortality during 1-4 years after birth. In addition, infant mortality is the largest contributor of under-five mortality (74.7%; 3760 thousands out of 5034 thousands).^3^ Therefore, this study shows critical evidence that mitigating the negative impact of drought on infant health would benefit the achievement of the Sustainable Development Goal 3.2 on reducing under-five child mortality. We now make this clear in the manuscript (lines 280-284 in the tracked version, lines 265-269 in the clean version):

*“Researchers have investigated the relationship between drought and under-five child mortality, with no plausible association observed. Compared to child mortality during 1-4 years after birth, infant mortality, the largest contributor of under-five mortality,^3^ is more likely to be biologically linked to drought exposure during pregnancy. However, there is still limited evidence on the negative impact of drought on infant deaths.”*

***Q3.*** *A part of the discussion is focused on the possible relationship between drought and diarrhea, did you consider to also study the possible links between drought and diarrhea? There are also questions about diarrhea in DHS.*

**A3.** Thank you for your comment. We actually published a paper on the relationship between drought and diarrhea among children. Please see here: <https://www.nature.com/articles/s41467-022-31291-7>.

***Q4.*** *Abstract. Overall the abstract summarizes the article in a good way. Results; the first sentence "The mean (standard deviation) number of months in which children experienced any, mild, or severe drought during pregnancy and survival period among cases was 0.371 (0.403), 0.263 57 (0.318), or 0.108 (0.245) respectively, and 0.370 (0.374), 0.267 (0.285), or 0.103 (0.216) respectively among non-cases". Difficult to get the meaning of this sentence, it is too much information, suggest to re-write and simplify.*

**A4.** We revised this sentence as below (lines 65-69 in the tracked version, lines 63-65 in the clean version):

*“The mean (standard deviation) number of months in which children experienced any drought during pregnancy and survival period was 4.6 (5.2) and 7.3 (7.4) among cases and non-cases, respectively.”*

***Q5.*** *Abstract. Conclusions: "Our findings urge more effective adaptation measures and alleviation strategies against the adverse impact of drought on child health." Should something about mitigation also be mentioned? I believe we need both adaptation and mitigation.*

**A5.** Thank you for your comment. You are correct that both adaptation and mitigation are urgently needed to minimize the negative impact of climate change on human health. However, the findings of this epidemiological study do not directly inform better mitigation strategies (i.e., lower carbon emission). Our results suggest important implications in improving infant health with better adaptation measures.

***Q6.*** *Main article. Introduction: Good. However, when reading it sounds like there is nothing else on infant/child mortality and drought in the literature, e.g. Delbiso et al, Drought and child mortality: a meta-analysis of small-scale surveys from Ethiopia, 2017, Scientific Reports and other similar studies are available.*

**A6.** Thank you for your comment and the information you provided. We are aware of the previous study on drought and child mortality. However, our study focused on drought and infant mortality, the mechanism of which could be different from the overall under-five mortality. Nevertheless, we added this information in our manuscript as below (lines 99-100 in the tracked version, lines 94-96 in the clean version):

*“To our knowledge, only one previous study in Ethiopia looked into the quantitative impact of drought on under-five child mortality, with no significant result reported.*^4^*”*

***Q7.*** *Main article. Methods: Overall well-described. Line 103; should "studies" be studied".*

**A7.** Thank you for noticing this typo. We have revised this accordingly in the manuscript (line 123 in the tracked version, line 119 in the clean version).

*“This region of the world deserves careful scrutiny, not only because the human health effects of climate change are infrequently studied among African populations, but also because it is one of the fastest growing populations, expected to be home to a quarter of the world’s population by 2050.”*

***Q8.*** *Main article. Methods: Line 120: "…we also collected data on ", sounds like the authors of this study collected the data? It is data collected by DHS.*

**A8.** We revised it as below (line 142 in the tracked version, line 138 in the clean version):

*“To compute a wealth index that is comparable across surveys, we also extracted data on household characteristics, including source of drinking water, type of toilet facilities, electricity, type of flooring, and possession of radio, television, phone (landline or cellphone), refrigerator, motorcycle, and car.”*

***Q9.*** *Main article. Results: Overall well-written, but a large focus on "significant results", especially the second last paragraph, line 233 to 241, there were a lot of analyses here and no surprise that some would be significant….*

**A9.** We have acknowledged this limitation of lack of adjusting for potential multiple comparison in our original manuscript (lines 351-354 in the tracked version, lines 336-339 in the clean version):

*“Fifth, in our secondary analysis we examined the associations between infant deaths during each of the 12 months after birth and drought exposure during each of the 21 months before and after birth, which might increase the possibility of chance associations due to multiple comparisons.”*

***Q10.*** *Main article. Discussions: Well-balanced. Line 267 - 268: "diarrhea, the second leading cause of death in children under five". This is true for the post-neonatal period, not overall under-five-mortality.*

**A10.** We now revise it as below (lines 298-299 in the tracked version, lines 283-284 in the clean version):

*“Drought has been found to be associated with an increased risk of diarrhea, one of the leading causes of death in children under five.”*

***Q11.*** *Main article. Discussions: Line 284-285: "Without any recognized direct impact…", we all know that humans of all ages need water to survive, so this is suggested to be re-written*

**A11.** Thank you for your suggestion. We have revised the sentence as below (lines 319-320 in the tracked version, lines 303-305 in the clean version):

*“Apart from the direct impact due to insufficient water supply, all indirect pathways linking drought to infant mortality contribute to the time lag in the association.”*

References:

1.	Ekholuenetale M, Wegbom AI, Tudeme G, Onikan A. Household factors associated with infant and under-five mortality in sub-Saharan Africa countries. *International Journal of Child Care and Education Policy.* 2020;14(1):10.

2.	Akinlo AE, Sulola AO. Health care expenditure and infant mortality in sub-Saharan Africa. *Journal of Policy Modeling.* 2019;41(1):168-178.

3.	United Nations Inter-agency Group for Child Mortality Estimation (UN IGME). *Levels & trends in child mortality: Report 2022, estimates developed by the United Nations Inter-agency Group for Child Mortality Estimation.* New York, 2023.

4.	Delbiso TD, Altare C, Rodriguez-Llanes JM, Doocy S, Guha-Sapir D. Drought and child mortality: a meta-analysis of small-scale surveys from Ethiopia. *Sci Rep.* 2017;7(1):2212.
