# Supplementary material for: Long-term drought and risk of infant mortality in Africa: A cross-sectional study
Source: PLoS Med. 2025 Jan 31;22(1):e1004516. doi: 10.1371/journal.pmed.1004516 (PMC11785314; doi:10.1371/journal.pmed.1004516)
Supplement: Attachment — Submitted filename: Response to reviewers_2nd.docx [file pmed.1004516.s011.docx]

**Response to editors and reviewers**

**Requests from the editors**

***Q1.*** *Data Availability: PLOS Medicine requires that the de-identified data underlying the specific results in a published article be made available, without restrictions on access, in a public repository or as Supporting Information at the time of article publication, provided it is legal and ethical to do so. PLOS defines the “minimal data set” to consist of the data set used to reach the conclusions drawn in the manuscript with related metadata and methods, and any additional data required to replicate the reported study findings in their entirety. Authors do not need to submit their entire data set, or the raw data collected during an investigation.*

*For each data source used in your study:*

*a) If the data are freely or publicly available, note this and state the location of the data: within the paper, in Supporting Information files, or in a public repository (include the DOI or accession number).*

*b) If the data are owned by a third party but freely available upon request, please note this and state the owner of the data set and contact information for data requests (web or email address). Note that a study author cannot be the contact person for the data.*

*c) If the data are not freely available, please describe briefly the ethical, legal, or contractual restriction that prevents you from sharing it. Please also include an appropriate contact (web or email address) for inquiries (again, this cannot be a study author).*

**A1.** We have a section about data availability in the manuscript (lines 400-404 in the tracked version, lines 397-401 in the clean version):

*“Data on infant mortality and socioeconomic status in this study were collected from the Demographic and Health Surveys program (*[*https://dhsprogram.com/*](https://dhsprogram.com/)*), which are freely available upon request. Publicly available meteorological records were obtained from the fifth generation ECMWF atmospheric reanalysis of the global climate (ERA5-Land, https://cds.climate.copernicus.eu/).”*

***Q2.*** *Financial Disclosure: The funding statement should include: specific grant numbers, initials of authors who received each award, URLs to sponsors’ websites. Also, please state whether any sponsors or funders (other than the named authors) played any role in study design, data collection and analysis, the decision to publish, or preparation of the manuscript. If they had no role in the research, include this sentence: “The funders had no role in study design, data collection and analysis, decision to publish, or preparation of the manuscript.*

**A2.** Now we revised our Funding section as below (lines 407-431 in the tracked version, lines 404-423 in the clean version):

*“PW, EOA, and KC received support from the Reckitt Global Hygiene Institute (https://rghi.org/; RGHI RIN: 2021-001). The views expressed are those of the authors and not necessarily those of RGHI. TR was funded by CTSA (Grant Number UL1 TR001863) from the National Center for Advancing Translational Science (NCATS; https://ncats.nih.gov/), a component of the National Institutes of Health (NIH). Its contents are solely the responsibility of the authors and do not necessarily represent the official views of NIH. JSR currently receives research support through Yale University from Johnson and Johnson to develop methods of clinical trial data sharing, from the Food and Drug Administration for the Yale-Mayo Clinic Center for Excellence in Regulatory Science and Innovation (CERSI; https://www.fda.gov/science-research/advancing-regulatory-science/centers-excellence-regulatory-science-and-innovation-cersis) program (U01FD005938), from the Agency for Healthcare Research and Quality (R01HS022882) (https://www.ahrq.gov/), and from Arnold Ventures; formerly received research support from the Medical Device Innovation Consortium as part of the National Evaluation System for Health Technology (NEST; https://www.fda.gov/about-fda/cdrh-reports/national-evaluation-system-health-technology-nest) and from the National Heart, Lung and Blood Institute (https://www.nhlbi.nih.gov/) of the National Institutes of Health (NIH) (R01HS025164, R01HL144644); and in addition, JSR was an expert witness at the request of Relator's attorneys, the Greene Law Firm, in a qui tam suit alleging violations of the False Claims Act and Anti-Kickback Statute against Biogen Inc. that was settled September 2022. The funders had no role in study design, data collection and analysis, decision to publish, or preparation of the manuscript.”*

***Q3.*** *Given your study is a observational cross-sectional study, Please ensure that the study is reported according to the STROBE guideline, and include the completed STROBE checklist as Supporting Information. Please add the following statement, or similar, to the Methods: ""This study is reported as per the Strengthening the Reporting of Observational Studies in Epidemiology (STROBE) guideline (S1 Checklist). When completing the checklist, please use section and paragraph numbers, rather than page numbers.*

*Did your study have a prospective protocol or analysis plan? Please state this (either way) early in the Methods section.*

*a) If a prospective analysis plan (from your funding proposal, IRB or other ethics committee submission, study protocol, or other planning document written before analyzing the data) was used in designing the study, please include the relevant prospectively written document with your revised manuscript as a Supporting Information file to be published alongside your study, and cite it in the Methods section. A legend for this file should be included at the end of your manuscript.*

*b) If no such document exists, please make sure that the Methods section transparently describes when analyses were planned, and when/why any data-driven changes to analyses took place.*

*c) In either case, changes in the analysis-- including those made in response to peer review comments-- should be identified as such in the Methods section of the paper, with rationale.*

*For all observational studies, in the manuscript text, please indicate: (1) the specific hypotheses you intended to test, (2) the analytical methods by which you planned to test them, (3) the analyses you actually performed, and (4) when reported analyses differ from those that were planned, transparent explanations for differences that affect the reliability of the study's results. If a reported analysis was performed based on an interesting but unanticipated pattern in the data, please be clear that the analysis was data-driven.*

**A3.** Our study is reported according to the STROBE guideline. And we now attach the STROBE checklist as a supplement material.

Our study did not have a prospectively registered protocol or analysis plan. We conducted the analysis following our statistical methodology pre-specified during lab meetings and over the informal communications among team members, and no data-driven changes to analyses took place during the process. We explicitly documented our study aims and detailed analytic strategies in the manuscript. We added one sentence in Methods (lines 234-237 in the tracked version, lines 232-235 in the clean version):

*“While this study did not have a prospectively registered protocol and statistical analysis plan, all variable definitions and analyses were pre-specified over the course of communications between the study team members and during laboratory meetings.”*

***Q4.*** *Statistical reporting: As per standard PLOS Medical requirements, please provide 95% CIs and p values for all results where appropriate (including the abstract). We suggest reporting statistical information in the following format: ‘x’; (95% CI [‘y’,’ z’] p value) and use commas as opposed to hyphens (as these can be confused with negative values) to separate upper and lower bounds. For p values, please report as p<0.001 and where higher as 'p=0.002'. Please add the statistical method used to your method section. We also invite you to report p values to consistently to the third decimal digit - thousandths.*

*If applicable, please include any important dependent variables that are adjusted for in the analyses.*

*Please include the actual amounts and/or absolute risk(s) of relevant outcomes (including NNT or NNH where appropriate), not just relative risks or correlation coefficients. (example for absolute risks: PMID: 28399126).*

**A4.** We have added the p values for all results, including the ones in the abstract. We also added one sentence in the method to show the significance level of our analysis (lines 233-234 in the tracked version, lines 231-232 in the clean version):

*“The significance level for all statistical tests was set at two-sided p<0.05.”*

The variables we adjusted in the models were all baseline characteristics and birth month and year, and we do not report any results for these variables in our manuscript. Our analysis does not yield any absolute risk.

***Q5.*** *Title: Please revise your title according to PLOS Medicine's style. Your title must be nondeclarative and not a question. It should begin with main concept if possible. "Effect of" should be used only if causality can be inferred, i.e., for an RCT. Please place the study design ("A randomized controlled trial," "A retrospective study," "A modelling study," etc.) in the subtitle (ie, after a colon).*

**A5.** We have revised our title as “The drier, the deadlier—the association between long-term drought and risk of infant mortality in Africa: A cross-sectional study.”

***Q6.*** *Abstract layout: Please structure your abstract using the PLOS Medicine headings (Background, Methods and Findings, Conclusions).*

**A6.** We have revised the headings accordingly.

***Q7.*** *Author summary: At this stage, we ask that you include a short, non-technical Author Summary of your research to make findings accessible to a wide audience that includes both scientists and non-scientists. The authors summary should consist of 2-3 succinct bullet points under each of the following headings:*

*Why Was This Study Done? Authors should reflect on what was known about the topic before the research was published and why the research was needed.*

*What Did the Researchers Do and Find? Authors should briefly describe the study design that was used and the study’s major findings. Do include the headline numbers from the study, such as the sample size and key findings.*

*What Do These Findings Mean? Authors should reflect on the new knowledge generated by the research and the implications for practice, research, policy, or public health. Authors should also consider how the interpretation of the study’s findings may be affected by the study limitations. In the final bullet point of ‘What Do These Findings Mean?’, please describe the main limitations of the study in non-technical language.*

**A7.** We now add the following author summary in the manuscript (lines 78-97 in the tracked version, lines 77-96 in the clean version):

*“Why was this done?*

*- Climate change is projected to result in a higher frequency and intensity of drought events.*

*- Previous studies have found rainfall anomaly to be associated with increased infant mortality.*

*- The risk of drought exposure for infant health outcomes is not well understood.*

*What did the researchers do and find?*

*- Employing an advanced measure of drought incorporating multiple climatic variables, we found that exposure to long-term severe drought was associated with increased risk of infant mortality in Africa.*

*- Severe drought exposure was significantly associated with a higher risk for neonatal mortality but not for postneonatal mortality.*

*- There is a delayed association between exposure to drought during pregnancy and risk of infant mortality.*

*What do these findings mean?*

*- The protection of pregnant women and young children from drought could potentially help reduce the heavy toll of infant mortality in Africa.*

*- Our study urges the prioritization of neonatal and pediatric care as well as the development of water, sanitation, and hygiene infrastructure and adaptation strategies in rural Africa.*

*- This study may underestimate the potential impact of drought on mortality in early life given it could have already contributed to miscarriage or stillbirth.”*

***Q8.*** *Introduction layout: Please address past research and explain the need for and potential importance of your study. Indicate whether your study is novel and how you determined that. If there has been a systematic review of the evidence related to your study (or you have conducted one), please refer to and reference that review and indicate whether it supports the need for your study.*

**A8.** In our manuscript we have explicitly described previous relevant research in this topic and indicated the potential research gap and therefore the necessity of this study.

Past research (lines 116-121 in the tracked version, lines 115-120):

*“To our knowledge, only one previous study in Ethiopia looked into the quantitative impact of drought on under-five child mortality, with no significant result reported. Furthermore, there is a paucity of evidence on the association between drought and infant mortality, although two studies from China and Brazil suggested such a link, in which drought exposure was characterized by rainfall fluctuations during the 12 months before birth.”*

The need for our study (lines 121-126 in the tracked version, lines 120-125):

*“Despite the lack of consensus on drought definition, there has been a growing recognition of the significance of incorporating temperature and other meteorological information, simultaneously taking water supply and demand into account when computing drought indices. However, previous studies only included precipitation in drought characterization and evidence on how such a more rigorously defined drought measure is associated with risk of infant deaths is missing.”*

Potential importance of our study (lines 133-135 in the tracked version, lines 132-134):

*“To date, there is a scarcity of research investigating the relationship between drought and infant mortality in Africa, a region that bears the greatest burden of infant deaths worldwide and where some profound drought events have occurred in the last century.”*

Indication of novelty (lines 144-148 in the tracked version, lines 143-146):

*“To fill these research gaps, by using data from 34 African countries and developing a complex drought indicator, we aimed to 1) examine the association between drought and risk of infant mortality; 2) investigate whether the association varies between neonatal and postneonatal deaths; and 3) explore the potential delayed effect of drought exposure on infant mortality.”*

There has been no systematic review relevant to our study.

***Q9.*** *Discussion layout: Please present and organize the Discussion as follows: a short, clear summary of the article's findings; what the study adds to existing research and where and why the results may differ from previous research; strengths and limitations of the study; implications and next steps for research, clinical practice, and/or public policy; one-paragraph conclusion.*

**A9.** The Discussion section is arranged in this fashion.

***Q10.*** *Supplementary materials: Please note that supplementary materials are not checked and will be posted as supplied by the authors. Therefore, please double check. Please cite your Supporting Information as outlined here: https://nam12.safelinks.protection.outlook.com/?url=https%3A%2F%2Fjournals.plos.org%2Fplosmedicine%2Fs%2Fsupporting-information&data=05%7C02%7Cpin.wang%40yale.edu%7Cb556ef45b23a4d34fd3108dc5fb85054%7Cdd8cbebb21394df8b4114e3e87abeb5c%7C0%7C0%7C638490492514333199%7CUnknown%7CTWFpbGZsb3d8eyJWIjoiMC4wLjAwMDAiLCJQIjoiV2luMzIiLCJBTiI6Ik1haWwiLCJXVCI6Mn0%3D%7C0%7C%7C%7C&sdata=7C8Yhuns6ZYrMbTaRtYpWuHPlZm6hyWzM%2FeMkuMGwpQ%3D&reserved=0 - Please note you may use almost any description as the item name of your supporting information as long as it contains an "S" and number. For example, “S1 Appendix” and “S2 Appendix,” “S1 Table” and “S2 Table. Please ensure each supplementary material has a call out (link) from your main manuscript.*

**A10.** We have re-arranged all supplementary materials using this style.

**Reviewer #3**

*Q1. Line 155-160: "In order to compute the SPEI at a timescale of 24 months (referred to as SPEI-24 hereinafter) for a given month during the study period, we first generated a time series by summing the water balance over the preceding 23 months up to the current month; then we transformed this series to a normal distribution with a mean of zero and standard deviation of one to eliminate the effect of both the local season and climate patterns."*

*This is still very confusing. I spent time reading the SPEI documentation. The documentation suggests that an essential step of fitting a probability distribution to the 24-month historical water balance data is missing here. This is integral for deriving the CDF used in the SPEI formula: SPEI = Z(CDF(WB)/(1-CDF(WB))). It is crucial to specify which probability distribution was selected to model the observed 24-month SPEI time series. Commonly, distributions such as the log-logistic, or Gamma, are employed. After fitting the appropriate distribution, the values are then transformed into a Z-score, reflecting a normal distribution with a mean of 0 and a standard deviation of 1. Given the prominence of SPEI as the major exposure metric in this study, it is imperative that the method of calculating this index is delineated with clarity and precision in the paper.*

**A1.** Thank you for your comment. According to the SPEI documentation (<https://climatedataguide.ucar.edu/climate-data/standardized-precipitation-evapotranspiration-index-spei>), the calculation of climatic water balance is based on a log-logistic distribution during the standardization process:

“Different analysis suggested selection of the Log-logistic distribution for standardizing the D series to obtain the SPEI since the Log-logistic distribution adapted very well to the D series for all time scales and climate regions. With very few exceptions, for most regions worldwide a good fit was found between the log-logistic distribution and Dk series, independent of the time scale (k) and the month of the year. This guarantees the robustness of SPEI calculations based on such probability distribution.”

We now revised our text as follows (lines 178-183 in the tracked version, lines 176-181 in the clean version):

*“In order to compute the SPEI at a timescale of 24 months (referred to as SPEI-24 hereinafter) for a given month during the study period, we first generated a time series by summing the water balance over the preceding 23 months up to the current month; then we transformed this series to a normal distribution with a mean of zero and standard deviation of one, according to a log-logistic distribution, to eliminate the effect of both the local season and climate patterns.”*

*Q2. "..There should be no such bias in the current analysis because our start of "follow-up" and start of exposure are both the start of pregnancy (time zero). Although each fetus had to survive through the entire pregnancy to be eligible to be included in the study sample,…"*

*What you're describing here aligns exactly with the concept of immortal time bias. In the context of your study, the fetus is 'immortal' from conception to birth, yet this period is still included as exposure time in the analysis. Actually, I believe the exposure period should start at birth, as fetal demise is not considered child mortality anyway. I would highly recommend you change the exposure start time for all the analyses in the paper.*

**A2.** Thank you for your comment.

Immortal time bias is caused by the misalignment between time zero (the time when participants enter the study) and treatment assignment, which, in our case, is the start of exposure. This usually happens when participants are assigned to exposed groups by using information that is observed AFTER time-zero (forward looking). Because these participants must have survived the time between time zero and exposure, they are considered “immortal”.

This means that, if immortal time bias is present, the start of exposure must be between time zero and outcome. However, in our study, our exposure starts from the start of pregnancy, and the time zero is birth (time zero is LATER than the start of exposure). This does not induce immortal time bias.

Immortal time bias can be commonly seen in perinatal epidemiology if one is investigating the relationship between pregnancy complications and stillbirth.^1,2^ Because pregnancy complications are not usually diagnosed until mid-to-late pregnancy, there can be a situation where pregnancies must “survive” a period of time in order to be screened for certain complications. In this case, time zero is conception, and treatment/exposure is the pregnancy complication, which is AFTER time zero. To reiterate, there was no immortal time bias in our study by design because our exposure status was NOT determined after the start of “follow-up” (time zero).

Instead, we run into another potential bias, namely live-birth bias.^2,3^ This bias may occur if both 1) drought increases the risk of miscarriage/stillbirths and 2) other factors (separate from drought) are associated with both the risk of miscarriage/stillbirth and infant mortality. One way of thinking of this would be that – in theory – fetuses lost due to drought may be the fetuses who would otherwise have been at the greatest risk of infant mortality (e.g., due to severe congenital malformations). Live-birth bias is generally thought to underestimate harmful effects on the offspring of environmental exposures during pregnancy. We therefore argue that including drought exposure during pregnancy is still reasonable, and if anything, the bias will be towards the null. We would also like to emphasize that exposures during the pregnancy period are often important for infant mortality, so it is highly relevant to also consider this period when possible.

We have acknowledged the limitation of this live birth bias in the Discussion section (lines 355-360 in the tracked version, lines 352-357):

*“First, despite a monthly measure, the calculated 24-month SPEI in our study incorporates multiple weather parameters to illustrate the overall dry condition for the preceding 24 months. However, drought and other unfavorable climatic conditions during this period could have already resulted in deaths in an earlier stage (i.e., miscarriage or stillbirth), introducing live-birth bias.^2^ This study focusing on infant mortality might potentially underestimate the detrimental impact of drought on deaths in early life.”*

Furthermore, the time-dependent analysis we adopted in this study is one of the most effective methods to minimize the impact of potential immortal time bias, if any.^2^

In addition, we performed another analysis in order to compare the association with exposure from the start of pregnancy through infant deaths (main analysis) and the exposure from birth through infant deaths (additional analysis). Please see the results below.

|  | Drought events | Estimate | Lower bound | Upper bound |
| --- | --- | --- | --- | --- |
| Main analysis | All drought | 1.019 | 0.998 | 1.040 |
|  | Mild drought | 1.011 | 0.988 | 1.034 |
|  | Severe drought | 1.040 | 1.008 | 1.074 |
| Additional analysis | All drought | 1.017 | 0.996 | 1.038 |
|  | Mild drought | 1.024 | 0.991 | 1.058 |
|  | Severe drought | 1.041 | 0.994 | 1.091 |

The estimated associations between infant deaths and drought exposure from birth and deaths are largely consistent with our main analysis, which further suggests the absence of immortal time bias as well as the robustness of our main analysis.

Moreover, one highlight of this study is the exploration of the potential delayed effect of drought exposure during pregnancy. Therefore, we would like to keep drought from the start of pregnancy through infant deaths as our exposure in all analyses.

References:

1. Hutcheon JA, Kuret V, Joseph KS, Sabr Y, Lim K. Immortal time bias in the study of stillbirth risk factors: the example of gestational diabetes. Epidemiology. 2013 Nov 1;24(6):787-90.

2. Neophytou AM, Kioumourtzoglou MA, Goin DE, Darwin KC, Casey JA. Educational note: addressing special cases of bias that frequently occur in perinatal epidemiology. International journal of epidemiology. 2021 Feb 1;50(1):337-45.

3. Liew Z, Olsen J, Cui X, Ritz B, Arah OA. Bias from conditioning on live birth in pregnancy cohorts: an illustration based on neurodevelopment in children after prenatal exposure to organic pollutants. International journal of epidemiology. 2015 Feb 1;44(1):345-54.
