# Supplementary material for: Long-term drought and risk of infant mortality in Africa: A cross-sectional study
Source: PLoS Med. 2025 Jan 31;22(1):e1004516. doi: 10.1371/journal.pmed.1004516 (PMC11785314; doi:10.1371/journal.pmed.1004516)
Supplement: Attachment — Submitted filename: Response to reviewers_3rd.docx [file pmed.1004516.s013.docx]

**Response to the reviewer**

*Reviewer #3: I am very confused by the authors' response regarding the survival bias, particularly concerning the time-zero of the study. Previously, the authors stated, "...There should be no such bias in the current analysis because our start of 'follow-up' and start of exposure are both the start of pregnancy (time zero)...". However, in the current response, they said, "...However, in our study, our exposure starts from the start of pregnancy, and the time zero is birth (time zero is LATER than the start of exposure)," which contradicts their earlier statement.

If time zero is indeed at birth, then the follow-up time should start at birth. In that case, there would be no survival bias, and that's why I suggested changing the time zero to birth (instead of the start of pregnancy) for their survival analyses. However, if time zero is the start of pregnancy, the period between the start of pregnancy and birth is immortal time. It seems the authors may not fully understand immortal bias. Immortal bias occurs when follow-up time is counted during a period when the outcome of interest cannot happen. Misalignment between time zero and treatment assignment can cause immortal bias, but this is not the definition of survival bias. If the authors start counting time from the the start of pregnancy, then there is survival bias. The authors seem to not fully understand the concepts, and I am very confused by their contradictory statements...

For clarification, here is a definition of time zero and follow-up time (time-to-event): "Time zero, or the time origin, is the time at which participants are considered at-risk for the outcome of interest... Follow-up time is measured from time zero (the start of the study or from the point at which the participant is considered to be at risk) until the event occurs, the study ends, or the participant is lost, whichever comes first."*

**Response:** Thank you very much for your insightful comment.

First, we want to sincerely apologize for our confusing statements about the time zero of this study. We totally agree with your comment that time zero is the time at which participants are considered at-risk for the outcome of interest. The crucial point is the timing of “at-risk”. Indeed, there is no “infant mortality” before a child is born. Therefore, every child is at risk of infant mortality starting from birth. In this sense, time zero should be birth.

However, the definitions of time zero and follow-up time in perinatal/child epidemiological studies have different implications from other epidemiological studies for adults. Newborns, infants, and children are indirectly affected by many types of prenatal exposure that directly impact the mother during pregnancy, such as temperature, air pollution, greenness, and medication, through the mother-fetus bond. It is because of this imperative link between the mother and child that there have been numerous epidemiological studies on the relationship between *in-utero* exposure and various birth/infant/child health outcomes. Please see some references below:

Guilbert A, Hough I, Seyve E, Rolland M, Quentin J, Slama R, Lyon-Caen S, Kloog I, Bayat S, Siroux V, Lepeule J. Association of prenatal and postnatal exposures to warm or cold air temperatures with lung function in young infants. JAMA network open. 2023 Mar 1;6(3):e233376.

Jung EM, Kim KN, Park H, Shin HH, Kim HS, Cho SJ, Kim ST, Ha EH. Association between prenatal exposure to PM2.5 and the increased risk of specified infant mortality in South Korea. Environment International. 2020 Nov 1;144:105997.

Amegbor PM, Sabel CE, Mortensen LH, Mehta AJ, Rosenberg MW. Early-life air pollution and green space exposures as determinants of stunting among children under age five in Sub-Saharan Africa. Journal of Exposure Science & Environmental Epidemiology. 2023 Jun 29:1-5.

Man KK, Chan EW, Ip P, Coghill D, Simonoff E, Chan PK, Lau WC, Schuemie MJ, Sturkenboom MC, Wong IC. Prenatal antidepressant use and risk of attention-deficit/hyperactivity disorder in offspring: population based cohort study. bmj. 2017 May 31;357.

We admit that due to this special perspective of “follow-up” time in perinatal studies, we did not fully comprehend previously how we should define time zero. We apologize again for making contradictory statements in our response letters. However, we argue that **maternal exposure to drought during pregnancy is potentially associated with infant mortality through the mother**, similar to other environmental exposures as stated before. In this sense, it is crucial for us to include the pregnancy period in our study. This is the primary and foremost reason why we used the start of pregnancy as the beginning of our analysis. Excluding the prenatal period would significantly jeopardize the quality of our study and inevitably raise questions about missing this critical exposure window. Furthermore, as we mentioned in our previous response letter, we would like to keep drought from the start of pregnancy through infant deaths as our exposure also because the exploration of the potential delayed effect of drought exposure during pregnancy is one of the highlights of our study.

Second, we also agree with the reviewer that “misalignment between time zero and treatment assignment can cause immortal bias, but this is not the definition of survival bias.” Therefore, we now acknowledge this immortal time bias in the Discussion section (lines 356-359):

*“First, our survival analysis started at the beginning of pregnancy, whereas our outcome of interest was infant mortality, which could not occur until birth. This misalignment may introduce immortal time bias, potentially overestimating or underestimating our investigated association.”*

In addition, as we mentioned previously, we performed another analysis to compare the association with exposure from the start of pregnancy through infant deaths (main analysis) and exposure from birth through infant deaths (additional analysis). We now add this analysis to the revised manuscript and supplementary information.

We revised our Methods as follows (lines 229-231):

*“Fourth, we performed an additional analysis to compare the association with exposure from the start of pregnancy through infant deaths and that with exposure from birth through infant deaths.”*

We revised our Results as follows (lines 287-289):

*“In addition, when excluding drought exposure during pregnancy, the association with severe drought was found to be non-significant (Figure S3).”*

According to the result of this sensitivity analysis, we revised our Figure S3 as follows:

The estimated associations between infant deaths and drought exposure from birth to death are largely consistent with our main analysis, which also suggests the robustness of our main analysis.

We hope that our explanation, acknowledgment of limitations, and additional analysis clarify this important issue you pointed out. We sincerely thank you again for your precious time in improving this study and manuscript.
