# Supplementary material for: Long-term drought and risk of infant mortality in Africa: A cross-sectional study
Source: PLoS Med. 2025 Jan 31;22(1):e1004516. doi: 10.1371/journal.pmed.1004516 (PMC11785314; doi:10.1371/journal.pmed.1004516)
Supplement: Attachment — Submitted filename: Response to reviewers_4th.docx [file pmed.1004516.s015.docx]

**Response to the editors and reviewers**

***Comments from editors:***

*1. We appreciate the inclusion of the potential for immortal time bias as a limitation of the study. However, we feel that some additional consideration is needed in terms of the statistical approach given the stated importance of the pregnancy period as part of the exposure (noting that the editors agree with the notion that exposure to draught conditions during pregnancy could affect the developing fetus) and in view of the statistician’s comment that this exposure has not been accounted for in the model.*
**Response:** Thank you for your comment. We have accounted for drought exposure during pregnancy in our models, which is exactly the discrepancy between reviewer 3 and us. We used the time-dependent variable for drought exposure from conception until infant death as our explanatory variable in the models. Please see our further response to the arbitrating reviewer 4 below.

*2. More detail is needed around the sensitivity analysis shown in figure S3, as noted by the arbitrating statistician.*
**Response:** We now report the full numerical results as Table S3 instead of Figure S3, to show the detailed comparison between the models.

**Table S3** Association between long-term drought and risk of infant mortality in various sensitivity analyses

|  | **Estimate** | **Standard error** | **Lower bound** | **Upper bound** |
| --- | --- | --- | --- | --- |
| **Main analysis** |  |  |  |  |
| Any drought | 1.019 | 0.010 | 0.998 | 1.040 |
| Mild drought | 1.011 | 0.011 | 0.988 | 1.034 |
| Severe drought | 1.040 | 0.016 | 1.008 | 1.074 |
| **Rainfall adjusted** |  |  |  |  |
| Any drought | 1.019 | 0.010 | 0.998 | 1.040 |
| Mild drought | 1.011 | 0.011 | 0.988 | 1.034 |
| Severe drought | 1.040 | 0.016 | 1.008 | 1.074 |
| **Birth month removed** |  |  |  |  |
| Any drought | 1.019 | 0.010 | 0.999 | 1.040 |
| Mild drought | 1.011 | 0.011 | 0.988 | 1.034 |
| Severe drought | 1.041 | 0.016 | 1.009 | 1.075 |
| **Complementary log-log regression** |  |  |  |  |
| Any drought | 1.013 | 0.007 | 0.998 | 1.027 |
| Mild drought | 1.003 | 0.008 | 0.988 | 1.019 |
| Severe drought | 1.038 | 0.011 | 1.015 | 1.061 |
| **Postnatal period only** |  |  |  |  |
| Any drought | 1.029 | 0.015 | 0.999 | 1.060 |
| Mild drought | 1.024 | 0.017 | 0.991 | 1.058 |
| Severe drought | 1.041 | 0.024 | 0.994 | 1.091 |

*3. Regarding the conclusions of the manuscript, I have discussed this with the editorial team, and we agree with the arbitrating statistical reviewer that the data presented in the paper do not support the strong conclusions. As noted by the statistician, in view of the results of the standard null hypothesis significance testing with a critical alpha of 0.05, the statement in the abstract should be amended to “Compared to children who did not experience drought, any drought exposure was *not* associated with an increased risk of infant mortality…” (or to the language suggested in the review). We also feel that the discordant result of the severe draught effect should be noted in the Abstract in the interest of full and transparent reporting.*
**Response:** According to Reviewer 4’s suggestion, we now modified the results section of the abstract as follows (lines 63-65):

*“Compared to children who did not experience drought, we did not find evidence that any drought exposure was associated with an increased risk of infant mortality (hazard ratio [HR]: 1.02, 95% confidence interval [CI] [1.00, 1.04], p=0.072).”*

In terms of the discordant result of severe drought effects between the main analysis and sensitivity analysis, we now revised the abstract as follows (lines 69-70):

*“However, when excluding drought exposure during pregnancy, the association with severe drought was found to be non-significant.”*

*4. The title of the manuscript should be changed to comply with PLOS Medicine style (it should not be declamatory) and to accurately reflect the data presented in the paper. We suggest “Long-term drought and risk of infant mortality in Africa: A cross-sectional study”.*
**Response:** We have revised the title as the editors suggested.

*5. We intend to send the revised paper to the statisticians for a final look. As noted above, it will be important for the paper to be acceptable from a statistical perspective for us to proceed to acceptance/publication.*

**Response:** We thank the efforts of the editors to improve the manuscript.

***Comments from reviewer #3:***
*I really appreciate the detailed explanations and the efforts the authors have made to revise the paper. However, I still do not agree with the decision to include the time between the start of pregnancy and the time of birth in the time-to-event outcome.
First, I fully agree with the authors' argument that "maternal exposure to drought during pregnancy is potentially associated with infant mortality through the mother." However, the time of exposure does not need to coincide with time-zero in a survival analysis. It can, but it does not have to. For example, you can examine the relationship between exposure to second-hand smoke before age 10 and "the risk of developing asthma after age 10." That's completely valid. However, the time-zero must start at age 10, not age 9, because the outcome is defined as "the risk of developing asthma <after> age 10." Any time before age 10 is "immortal time" because, by definition, it is impossible for the person to have the outcome.
For infant mortality, by definition, it is the death of a newborn <after birth> and before age 1. Therefore, "infant mortality" cannot occur before birth. Please note that the concept of time-zero in a survival analysis is related to the outcome (i.e., time to the event, which is infant mortality) rather than to the exposure. I believe the authors are still confused on this point.
It is encouraging to hear that using the time of birth as time-zero does not significantly change the results (in fact, by removing the immortal time period, I would expect the relationship to be stronger). Therefore, I recommend reporting those results using the time of birth as time-zero. Why do we have to introduce immortal time bias when it can be avoided?*

**Response:** We first would like to thank you for the thoughtful comments and efforts to help us improve the manuscript.

After a thorough discussion with all co-authors, we still don't agree with you regarding the change of time zero from conception to birth. Although it can avoid immortal time bias, as we mentioned in the response letter, excluding the prenatal period would significantly jeopardize the quality of our study and inevitably raise questions from other researchers about missing this critical exposure window. In our last response letter, we have listed a number of papers published in other prestigious journals that also incorporated the pregnancy period in the analysis. Furthermore, the employment of time-dependent survival analysis itself is one way to minimize the immortal time bias.

Also, in an article posted by Columbia University School of Public Health, we learn that "time origins can also be determined by a defining characteristic, such as onset of exposure or diagnosis. This is often a natural choice if the outcome is related to that characteristic" (https://www.publichealth.columbia.edu/research/population-health-methods/time-event-data-analysis#:~:text=The%20time%20origin%20is%20the,most%20commonly%20time%20on%20study).

Nevertheless, in response to the your comment and to ensure that both approaches provided largely consistent findings, we conducted sensitivity analyses using the methods suggested by the reviewer. As seen in the Figure S3 (now Table S4), our results are broadly consistent. Because we continue to believe that accounting for the in-utero exposure period is critical for investigating the health effects of drought on infant mortality, we have retained that approach as our primary analysis. But we now report the sensitivity analysis to demonstrate that our results are robust to either approach.

We do think that the acknowledgment of potential immortal time bias is much more important than the exclusion of the pregnancy period in this study, from an epidemiologist's perspective.

***Comments from reviewer #4:***
*This paper examines the association between drought and infant mortality in Africa.*

*The statistical issue*

*There is no dispute that the Cox model is appropriate and also the logistic model as outlined in S1 Method. The issue turns on when time is taken to start. The authors start time at conception. Since that is unobservable they impute it as occurring nine months before birth. Obviously this rests on assumptions but you have to make some assumptions in life to get any- where and this imputation has not been disputed. The statistical reviewer by contrast takes the view that since the outcome is infant mortality the time should start at birth since it is only then that the child is at risk of infant mortality. My initial response would have been that I agreed with the reviewer as I cannot see why one would want to include time when the participants were not at risk of the event in a time to event model. The authors have responded that*

*We apologize again for making contradictory statements in our response letters. However, we argue that maternal exposure to drought during pregnancy is potentially associated with infant mortality through the mother, similar to other environmental exposures as stated before. In this sense, it is crucial for us to include the pregnancy period in our study. This is the primary and foremost reason why we used the start of pregnancy as the beginning of our analysis. Excluding the prenatal period would significantly jeopardize the quality of our study and inevitably raise questions about missing this critical exposure window. Furthermore, as we mentioned in our previous response letter, we would like to keep drought from the start of pregnancy through infant deaths as our exposure also because the exploration of the potential delayed effect of drought exposure during pregnancy is one of the highlights of our study.*

*I am not an expert in human development but it seem obvious that adverse circumstances during pregnancy would affect the mother–to–be’s chances of producing a thriving infant. Those circumstances seem to me to be an exposure, in the terminology of the equation in S1 Method they would be in the xi2. As I read the authors’ rebuttal quoted above it seems to me that although they agree that it is an exposure they seem unwilling that it should be treated as such. As it stands I do not see how the goal of assessing its effect is being met by the authors’ analysis.*

*The sensitivity analyses*

*The authors have fitted the model as suggested by the reviewer as one of their sensitivity analyses and report it on page 12.*

*In addition, when excluding drought exposure during pregnancy, the association with severe drought was found to be non-significant (Figure S3).*

*This is insufficient. Just showing a graph without any numerical values does not allow us to make a proper comparison between the models. Looking at figure S3 it seems (using a ruler) that the confidence intervals are somewhat wider. My intuition here would be that excluding irrelevant time periods might have given more precise estimates, not less. The reviewer had already suggested this.*

*The conclusions*

*In the abstract we read*

*Compared to children who did not experience drought, any drought exposure was associated with an increased risk of infant mortality (hazard ratio [HR]: 1.02, 95% confidence interval [CI] [1.00, 1.04], p=0.072).*

*Given the findings of the two models this seems to me to be far too strong. The authors are adopting the standard null hypothesis significance test (NHST) model and state on page 10 that their critical α is 0.05. Given that the statement in the abstract should read ”Compared to children who did not experience drought, we were unable to find evidence that any drought exposure was associated with an increased risk of infant mortality (hazard ratio [HR]: 1.02, 95% confidence interval [CI] [1.00, 1.04], p=0.072).” In fact given the narrowness of the confidence intervals one could stretch a point and state there is evidence of no effect. That would also make clear that both the authors’ preferred analysis and that of the statistical reviewer concur in the scientific finding. The discordance of the finding about severe drought should remain. Some corresponding changes would be needed elsewhere. In particular the title ‘The drier, the deadlier’ is not what was found by the authors.*

*Recommendations*

*•	Provide full details of the sensitivity models (ideally all of them not just the one using time since birth).*

*•	Change the abstract as I have suggested so as to comply with NHST.*

*•	Since the two models agree on all the substantive issue except severe drought there is no pressing need to swap the roles of primary analysis and sensitivity analysis.*

*Obviously the fact that the authors did not find any evidence of effect has no relevance on the decision to publish or not.*

**Response:** We highly appreciate your detailed comments, which significantly help solve the dispute and improve the manuscript.

The statistical issue:

We totally agree that, as you pointed out, “*adverse circumstances during pregnancy would affect the mother-to-be's chances of producing a thriving infant. Those circumstances seem to me to be an exposure.*” This is the principal reason why we insist on including the pregnancy period in our main analysis. It is reasonable to hypothesize that environmental exposure during pregnancy has an adverse effect on infants, as we have seen from numerous previous environmental health studies. Therefore, we have to respectfully disagree with your comment “*although they agree that it is an exposure they seem unwilling that it should be treated as such.*” On the contrary, drought during pregnancy is an exposure we strongly insist on in our analysis from the very beginning, which is exactly the dispute between reviewer 3 and us.

You also mentioned “*As it stands I do not see how the goal of assessing its effect is being met by the authors' analysis.*” In our main analysis, we used a time-dependent variable representing monthly drought conditions as our explanatory variable in the model (with or without drought for each month). By incorporating it into the Cox model, we can evaluate the potentially harmful effect of drought exposure from the start of pregnancy until infant death.

In addition, you mentioned *“Those circumstances seem to me to be an exposure, in the terminology of the equation in S1 Method they would be in the xi2.”* We believe that “those circumstances” refer to adverse circumstances during pregnancy. However, as is written in the supplement, *x_i2_* represents time-invariant covariates, including child’s sex, area of residence, mother’s education, and wealth quintile (e.g., not including an indicator of pregnancy [yes/no]). To further clarify, **we did not include any variable representing the pregnancy period itself**. Instead, to reiterate, we used a time-varying indicator for drought exposure, represented by *x_i_*_1_(*t*) in the equation, from conception until infant death, to assess the association between monthly drought conditions and infant mortality.

To conclude, we feel that there might be some misunderstanding of the statistical method of our main analysis in terms of how we evaluated the potential impact of drought exposure **during pregnancy**. We did not use an indicator for pregnancy itself but used a time-dependent variable to account for monthly drought exposure before and after birth.

The sensitivity analyses:

We agree with you that there is a necessity to report more appropriate results to compare the results between models. Now we include Table S3, instead of Figure S3, to show all numerical values of all sensitivity analyses as well as our primary analysis.

However, from either Table S3 or Figure S3, we can see that after excluding the pregnancy period (please see the results under “postnatal period only”), **the confidence intervals are actually wider than the results from our main analysis**. And the association with severe drought became non-significant in this sensitivity analysis. This further demonstrates the importance of the **inclusion** of the pregnancy period in the analysis, which gave **more precise** estimates, not less. Therefore, pregnancy is not irrelevant but a critical time period we must take into account.

The conclusions:

We also agree with you that with our current hypothesis testing, our conclusions might be too strong. We now revised our abstract as follows (lines 63-65):

*“Compared to children who did not experience drought, we did not find evidence that any drought exposure was associated with an increased risk of infant mortality (hazard ratio [HR]: 1.02, 95% confidence interval [CI] [1.00, 1.04], p=0.072).”*

However, we also respectfully disagree with you that “there is evidence of no effect.” There indeed is a positive association between any drought exposure and infant mortality, although it was only borderline significant.

In addition, in the interest of a full and transparent report, we also added an additional sentence in the abstract to show the discordant findings about severe drought between our main and sensitivity analysis (lines 69-70).

*“However, when excluding drought exposure during pregnancy, the association with severe drought was found to be non-significant.”*

According to your comments, we also changed our manuscript title to “Long-term drought and risk of infant mortality in Africa: A cross-sectional study.”

We hope that our responses will help solve this important issue. We wanted to sincerely thank the editors and reviewers again for your precious time in improving this study and manuscript.
