# Supplementary material for: Long-term drought and risk of infant mortality in Africa: A cross-sectional study
Source: PLoS Med. 2025 Jan 31;22(1):e1004516. doi: 10.1371/journal.pmed.1004516 (PMC11785314; doi:10.1371/journal.pmed.1004516)
Supplement: Attachment — Submitted filename: Response to reviewers_5th.docx [file pmed.1004516.s016.docx]

**Response to editor and reviewer**

***To the editorial team:***

After discussion with the editorial team, we see that there is a need to further clarify the main model, especially for non-environmental epidemiologists and physicians. Now we have revised the first paragraph of our statistical analysis as follows (lines 192-203):

*“We used an extended Cox regression model with time-dependent covariates to examine the association between drought at a timescale of 24 months and risk of infant mortality. Previously applied in other infant death studies,^1^ this extended model allows for time-varying covariance caused by the change of a given covariate over time during the follow-up period.^2^ It is necessary to reiterate that our explanatory variable, the monthly drought indicator, changed over time (indicating the presence or absence of drought conditions for each month from conception to infant death). Our time-dependent Cox regression analysis was an appropriate approach for handling the time-varying drought indicator because, during each month in which an infant death occurred, the model compared the current drought condition experienced by the deceased infant with the current drought conditions experienced by all other infants at risk at that time.^3^ With its ability to incorporate time-varying covariates, this method has been widely adopted in both environmental and non-environmental epidemiological research.^1,4^”*

***To Dr. Dewey:***

We thank Dr. Dewey very much for your important comments. We would like to respond to your second comment first.

*Comparing the models*

*The authors state in their rebuttal “This further demonstrates the importance of the inclusion of the pregnancy period in the analysis, which gave more precise estimates, not less.” Exactly. That is why in my review I stated “Those circumstances seem to me to be an exposure, in the terminology of the equation in S1 Method they would be in the xi2.” The authors have not, as far as I can see, included this. If they truly believe exposure in pregnancy is important they should have included it in the sensitivity analysis as I suggested, as an exposure, an xi2 and then reported the coefficient.*

**Response:** Thank you for clarifying this. We apologize that there was some misunderstanding in our correspondence last time. We stated, “we did not include any variable representing the pregnancy period itself,” because we thought that you suggested we put a binary indicator of pregnancy period in the model. That is why we reiterated our analytic approach in our last response letter.

Here, we want to share our data structure first. Please see the screenshot below. This is a child who died in their 8^th^ month after birth. The “endpt” means the death event and “if_dry24_all” represents if there is any drought condition in a relevant month (“if_dry24_md” and “if_dry24_sv” represent mild and severe drought respectively). As you can see, there is a data point for drought exposure in each month from conception to death.

In our primary analysis, the exposure during pregnancy has been taken into account by the time-varying covariate of drought in the model. In the sensitivity analysis, we excluded the pregnancy period by dropping the rows of “tstart” from 1 through 9.

As you suggested, we performed another sensitivity analysis on top of the previous sensitivity analysis that the other reviewer suggested, incorporating exposure during pregnancy in the model. However, since there are nine drought indicators during pregnancy for each child, we computed two alternative variables to assess the effect of gestational drought:

1. the total number of drought months a child experienced during pregnancy, which was included in the model as a continuous variable.
2. if a child ever experienced any drought month during pregnancy, which was included in the model as a binary variable.

We now report their coefficients and uncertainties in Table S4 in the supplementary information, together with the associations with drought exposure after birth in these models. Please note that mild and severe drought were in the same model (using a multilevel variable).

**Table S4** Estimates and uncertainties of drought exposure before and after birth when including gestational drought exposure in the sensitivity analysis of postnatal period only

|  | **Estimate** | **Standard error** | **Lower bound** | **Upper bound** |  |  |  |  |
| --- | --- | --- | --- | --- | --- | --- | --- | --- |
| Number of drought months before birth |  |  |  |  |  |  |  |  |
| Any drought model | 1.0063 | 0.0023 | 1.0018 | 1.0108 |  |  |  |  |
| Mild & severe drought model^†^ | 1.0062 | 0.0023 | 1.0017 | 1.0107 |  |  |  |  |
| Drought exposure after birth |  |  |  |  |  |  |  |  |
| Any drought | 1.0054 | 0.0173 | 0.9719 | 1.0400 |  |  |  |  |
| Mild drought | 1.0035 | 0.0183 | 0.9682 | 1.0402 |  |  |  |  |
| Severe drought | 1.0114 | 0.0141 | 0.9610 | 1.0644 |  |  |  |  |
| Any drought month before birth | | | | |  |  |  |  |
| Any drought model | 1.0402 | 0.0166 | 1.0068 | 1.0747 |  |  |  |  |
| Mild & severe drought model | 1.0396 | 0.0167 | 1.0062 | 1.0742 |  |  |  |  |
| Drought exposure after birth |  |  |  |  |  |  |  |  |
| Any drought | 1.0122 | 0.0166 | 0.9798 | 1.0457 |  |  |  |  |
| Mild drought | 1.0088 | 0.0179 | 0.9741 | 1.0448 |  |  |  |  |
| Severe drought | 1.0219 | 0.0250 | 0.9729 | 1.0733 |  |  |  |  |

^†^ In this model, mild and severe drought conditions were represented by a three-level categorical indicator (no drought, mild drought, and severe drought).

We can see that, in the sensitivity analysis starting from birth, after including drought exposure during pregnancy, the association with drought after birth considerably attenuated (compared to the sensitivity analysis without drought exposure during pregnancy, Table S3). **However, the association with drought exposure before birth, represented by either indicator, is statistically significant. This additional analysis further demonstrated the importance of including drought exposure during pregnancy in the primary analysis.**

We also revised our manuscript regarding the statistical analysis and results as follows:

Statistical analysis (lines 239-245):

*“In addition, to test the importance of including gestational exposure in our main model, we incorporated drought during pregnancy in the fourth sensitivity analysis starting from birth. Since there were nine drought indicators during pregnancy for each child, we computed two alternative variables to assess gestational drought in the models: 1) an indicator of the total number of drought months a child experienced during pregnancy as a continuous variable, and 2) an indicator of any drought month a child ever experienced during pregnancy as a binary variable.”*

Results (lines 301-306):

*“In addition, when we started the analysis from birth, the association with severe drought was found to be non-significant (Table S3). However, when we included drought exposure during pregnancy as a covariate in this postnatal-period-only analysis, it showed a statistically significant association with our outcome (Table S4), further demonstrating the importance of including this prenatal exposure to drought in our primary analysis.”*

*Conclusions in the abstract*

*The authors state “However, when excluding drought exposure during pregnancy, the association with severe drought was found to be non-significant” and “Exposure to long-term severe drought was associated with increased infant mortality risk in Africa.” These contradict one another. The authors have fitted two different models, their preferred one and the one suggested by the statistical reviewer. Each gives three views of the data: overall, severe and not severe. Only one of the six showed a difference. The conclusion is still too strong.*

**Response:** First, we have been very transparent about our results by including the sensitivity analysis in the abstract:

*“However, when excluding drought exposure during pregnancy, the association with severe drought was found to be non-significant.”*

Indeed, we fitted two different models, but they have different roles. The model with pregnancy period (“our preferred one”) was the **primary analysis** model, while the model excluding pregnancy period (“the one suggested by the statistical reviewer”) was the **sensitivity analysis** model. The primary analysis is the one we rely on to draw our conclusions, and the sensitivity analysis is the one we use to test our models’ robustness or compare results across different models.

Furthermore, in our updated sensitivity analysis that you suggested, we found that drought exposure during pregnancy, represented by either indicator we computed, was statistically significantly associated with infant death. This result further showed that it was necessary to include this prenatal exposure in the primary analysis.

To reiterate, the choice of primary or sensitivity analysis was based on our research question and overarching consideration of the potential impact of exposure during pregnancy.

Yes we reported three results based on any, mild, and severe drought conditions from the primary analysis. And yes only severe drought showed a significant association. That is why, in the conclusion of the abstract, we state, “Exposure to long-term **severe** drought was associated with increased infant mortality risk in Africa.” We only focus on severe drought for the conclusion. And this conclusion is drawn from our primary analysis, not from the sensitivity analysis. In this sense, our conclusion is precise and appropriate.

Regarding the difference in the association with severe drought between the primary and sensitivity analysis, their point estimates were quite close (1.040 vs. 1.041), but with different uncertainties. We did not use the term “significantly” in the conclusion, which we have already taken into account the difference between the models. However, it is undeniable that even in the sensitivity analysis, there was a positive association, although it was not statistically significant.

Most importantly, since we have described in the manuscript very clearly how we performed our primary analysis, together with the findings from the new sensitivity analysis, this conclusion precisely reflected what we found from our primary analysis.

*Summary*

*The authors seem very reluctant to change anything in the light of the very real concerns that I and the other statistician raised.*

**Response:** We apologize that you feel that we are uncomfortable making changes to our study and manuscript. However, during the 5-round revisions, based on your and other reviewers’ comments and suggestions, we have significantly revised our manuscript and added several important components to our manuscript, including the addition of sensitivity analysis removing the pregnancy period, further including prenatal exposure in this sensitivity analysis, its relevant numeric outputs, and the addition of limitation about the immortal time bias, among others. We truly respect and appreciate the time and effort that you and other reviewers have put into this work and, therefore, have made changes that we feel necessary to address the reviewers’ concerns without compromising our research philosophy. Please bear with us if we don’t entirely agree with each and every comment of yours and others.

Regarding the “very real concerns” you mentioned – immortal time bias, we have demonstrated this in our previous responses:

*“Because we continue to believe that accounting for the in-utero exposure period is critical for investigating the health effects of drought on infant mortality, we have retained that approach as our primary analysis. But we now report the sensitivity analysis to demonstrate that our results are robust to either approach. We do think that the acknowledgment of potential immortal time bias is much more important than the exclusion of the pregnancy period in this study, from an epidemiologist's perspective.”*

We understand that there can be discrepancies between biostatisticians and epidemiologists in terms of how the statistical analysis should be performed to answer the research question and how the findings should be interpreted. For example, the other statistician insisted on dropping the pregnancy period to address the issue of immortal time bias. However, we, as epidemiologists, insisted on including this important period and acknowledging this limitation. Another example is that in our previous rebuttal letter, we stated:

*“However, we also respectfully disagree with you that “there is evidence of no effect.” There indeed is a positive association between any drought exposure and infant mortality, although it was only borderline significant.”*

We don’t want to rely on the statistical significance only to reveal our findings. A non-significant, especially borderline significant, positive association is definitely something worthy of reporting.

As we emphasized before, the potential exposure during pregnancy is critically important in examining its association with adverse birth outcomes and child health. This is why we always stand firm in including the pregnancy period in our **primary analysis** during the entire revision cycle. However, we acknowledge the potential existence of immortal time bias in our design. Please note that given the necessity of inclusion of the pregnancy period in the analysis, the immortal time bias issue is not solvable. Accordingly, we first stated this in the manuscript as the very first and foremost limitation of this study. Second, we performed the sensitivity analysis to compare our results.

In conclusion, we have tried our best to accommodate the comments and suggestions of all reviewers, because we fully understand that we all are working toward the same goal – a better study and publication.

**References**

1.	Platt RW, Joseph KS, Ananth CV, Grondines J, Abrahamowicz M, Kramer MS. A proportional hazards model with time-dependent covariates and time-varying effects for analysis of fetal and infant death. *Am J Epidemiol*. Aug 1 2004;160(3):199-206. doi:10.1093/aje/kwh201

2.	Zhang Z, Reinikainen J, Adeleke KA, Pieterse ME, Groothuis-Oudshoorn CGM. Time-varying covariates and coefficients in Cox regression models. *Ann Transl Med*. Apr 2018;6(7):121. doi:10.21037/atm.2018.02.12

3.	Therneau T, Crowson C, Atkinson E. Using time dependent covariates and time dependent coefficients in the Cox model. Accessed Oct 26, 2024. <https://cran.r-project.org/web/packages/survival/vignettes/timedep.pdf>

4.	Lepeule J, Rondeau V, Filleul L, Dartigues JF. Survival analysis to estimate association between short-term mortality and air pollution. *Environ Health Perspect*. Feb 2006;114(2):242-7. doi:10.1289/ehp.8311
