# Supplementary material for: Long-term drought and risk of infant mortality in Africa: A cross-sectional study
Source: PLoS Med. 2025 Jan 31;22(1):e1004516. doi: 10.1371/journal.pmed.1004516 (PMC11785314; doi:10.1371/journal.pmed.1004516)
Supplement: Attachment — Submitted filename: Response to editors_6th.docx [file pmed.1004516.s017.docx]

**Response to editors**

Q1. Abstract, line 67-68: please amend the wording from “…and a non-significant association with mild drought” to “but no significant association with mild drought”.

A1. We have revised the statement as follows (lines 66-69):

*“When stratified by drought severity, we found a statistically significant association with severe drought (HR: 1.04; 95% CI [1.01, 1.07], p=0.015) but no significant association with mild drought (HR: 1.01; 95% CI [0.99, 1.03], p=0.353), compared to non-exposure to any drought.”*

Q2. Author summary: for full transparency, please include a bullet point to report the overarching finding that there was no evidence that any drought exposure was associated with an increased risk of infant mortality. It would make sense for this to precede the point about the association with severe drought.

A2. We have revised the second section of Author Summary as follows (lines 86-93):

*“What did the researchers do and find?*

*- Employing an advanced measure of drought incorporating multiple climatic variables, we found no evidence that any drought exposure was associated with an increased risk of infant mortality in Africa.*

*- When stratifying the analysis by drought severity, exposure to long-term severe drought was observed to be associated with higher infant mortality risk.*

*- There is a delayed association between exposure to drought during pregnancy and risk of infant mortality.”*

Q3. Methods, line 163: please insert “et al” after the author name (Bendavid).

A3. We revised the sentence as follows (lines 166-168):

*“Following the method described by Bendavid et al. [35], we then performed a principal component analysis, generated a wealth index, and employed the quintiles of this index in the subsequent analysis.”*

Q4. Results, line 276-278: please rephrase this sentence so that it’s consistent with the abstract. Ie, “Compared to children who did not experience drought, we did not find evidence that any drought exposure was associated with an increased risk of infant mortality (hazard ratio [HR]: 1.02; 95% confidence interval [CI] [1.00, 1.04], p=0.072).”

A4. We revised it as follows (lines 279-281):

*“Compared to children who did not experience drought, we did not find evidence that any drought exposure was associated with an increased risk of infant mortality (hazard ratio [HR]: 1.02; 95% confidence interval [CI] [1.00, 1.04], p=0.072) (Figure 2).”*

Q5. Results, line 281-283: please modify the following sentence to report lack of an association rather than a non-significant association. “A stronger but non-significant association with mild drought was estimated for postneonatal mortality (HR: 1.02; 95% CI [0.99, 1.06], p=0.203) than for neonatal mortality (HR: 0.99; 95 % CI [0.96, 1.02], p=0.657).”

A5. We have revised the sentence as follows (lines 284-286):

*“This null association with mild drought was estimated for both postneonatal mortality (HR: 1.02; 95% CI [0.99, 1.06], p=0.203) and neonatal mortality (HR: 0.99; 95 % CI [0.96, 1.02], p=0.657).”*

Q6. Discussion: For completeness, we ask that you report the topline finding of no association between any drought exposure and infant mortality, as part of the initial paragraph of the Discussion.

A6. We revised the initial paragraph of Discussion as follows (lines 311-316):

*“In this cross-sectional, multi-country study, we used an advanced drought measure and found no evidence that any drought exposure was associated with an increased risk of infant mortality in African LMICs. However, long-term severe drought exposure was observed to be significantly associated with a higher infant mortality risk. A significantly stronger association was observed among children living in rural areas than among those in urban areas, and among children living in tropical zones compared to those in dry zones.”*

Q7. References: please format the references according to PLOS style. In the text, references should be cited in square brackets (e.g., “We used the techniques developed by our colleagues [19] to analyze the data.” Full details on reference formatting can be found here: <https://journals.plos.org/plosmedicine/s/submission-guidelines#loc-references>.

A7. We have formatted the in-text citation and reference list according to the PloS style.

Q8. Please remove the data availability statement from the main paper; this information will be included with the article meta-data (completed as part of the submission process).

A8. The data availability statement has been removed from the manuscript.

Q9. Figure 1: Please confirm that the appropriate usage rights apply to the use of this map. Please see our guidelines for map images: <https://journals.plos.org/plosmedicine/s/figures#loc-maps>

A9. The base map we used was obtained from ArcGIS Hub (<https://hub.arcgis.com/datasets/07610d73964e4d39ab62c4245d548625/>). According to the description of the “custom license,” this map data is available to the public without constraint.

Q10. Figure 2: please define all elements of box plots in the figure caption - center line, box limits and whiskers.

A10. Figure 2 is not a set of box plots but a group of error bar charts. We have explicitly stated this in the figure caption:

*“****Figure 2*** *Error bar charts for the associations between risk of infant mortality and exposure to long-term drought represented by 24-month standardized precipitation and evapotranspiration index by drought severity (any, mild, or severe) and type of infant mortality (neonatal or postneonatal mortality).”*

Q11. The supplemental file appears not to have been included in the most recent revision (these files are unfortunately not carried over from one revision to the next). Please ensure that a final version of the supplement is uploaded with this final revision.

A11. We have now uploaded the final version of the supplemental file.

In addition, this study was conducted when I was working at Yale University School of Public Health. However, now I have a new position at the University of Maryland School of Public Health. Therefore, in this revision, I added a new affiliation for myself and changed the primary email address for corresponding. Thank you for your understanding and accommodation for this change!
